# Environmental Persistence of the World's Most Burdensome Infectious and Parasitic Diseases

**DOI:** 10.3389/fpubh.2022.892366

**Published:** 2022-07-08

**Authors:** Skylar R. Hopkins, Isabel J. Jones, Julia C. Buck, Christopher LeBoa, Laura H. Kwong, Kim Jacobsen, Chloe Rickards, Andrea J. Lund, Nicole Nova, Andrew J. MacDonald, Miles Lambert-Peck, Giulio A. De Leo, Susanne H. Sokolow

**Affiliations:** ^1^National Center for Ecological Analysis and Synthesis, Santa Barbara, CA, United States; ^2^Department of Applied Ecology, North Carolina State University, Raleigh, NC, United States; ^3^Hopkins Marine Station, Stanford University, Pacific Grove, CA, United States; ^4^Department of Biology and Marine Biology, University of North Carolina Wilmington, Wilmington, NC, United States; ^5^Department of Epidemiology, Stanford University, Stanford, CA, United States; ^6^Stanford Woods Institute for the Environment, Stanford University, Stanford, CA, United States; ^7^School of Veterinary Medicine, University of California, Davis, Davis, CA, United States; ^8^Department of Ecology and Evolutionary Biology, University of California, Santa Cruz, Santa Cruz, CA, United States; ^9^Emmett Interdisciplinary Program in Environment and Resources, Stanford University, Stanford, CA, United States; ^10^Department of Biology, Stanford University, Stanford, CA, United States; ^11^Earth Research Institute, University of California, Santa Barbara, Santa Barbara, CA, United States; ^12^United Nations University for the Advanced Study of Sustainability, Tokyo, Japan; ^13^Marine Science Institute, University of California, Santa Barbara, Santa Barbara, CA, United States

**Keywords:** environmental control, DALYs, disease dynamics, human health, human–environment interaction

## Abstract

Humans live in complex socio-ecological systems where we interact with parasites and pathogens that spend time in abiotic and biotic environmental reservoirs (e.g., water, air, soil, other vertebrate hosts, vectors, intermediate hosts). Through a synthesis of published literature, we reviewed the life cycles and environmental persistence of 150 parasites and pathogens tracked by the World Health Organization's Global Burden of Disease study. We used those data to derive the time spent in each component of a pathogen's life cycle, including total time spent in humans versus all environmental stages. We found that nearly all infectious organisms were “environmentally mediated” to some degree, meaning that they spend time in reservoirs and can be transmitted from those reservoirs to human hosts. Correspondingly, many infectious diseases were primarily controlled through environmental interventions (e.g., vector control, water sanitation), whereas few (14%) were primarily controlled by integrated methods (i.e., combining medical and environmental interventions). Data on critical life history attributes for most of the 150 parasites and pathogens were difficult to find and often uncertain, potentially hampering efforts to predict disease dynamics and model interactions between life cycle time scales and infection control strategies. We hope that this synthetic review and associated database serve as a resource for understanding both common patterns among parasites and pathogens and important variability and uncertainty regarding particular infectious diseases. These insights can be used to improve systems-based approaches for controlling environmentally mediated diseases of humans in an era where the environment is rapidly changing.

## Introduction

The global burden of human infectious diseases has declined by more than 40% since the turn of the 21^st^ century (1), but long-term control efforts for some diseases have been stymied by agricultural intensification, urbanization, and other drivers of global change (2, 3). Changes to socio-ecological systems affect human interactions with the abiotic and biotic environment, thereby modifying contact between susceptible humans and parasites and pathogens with environmentally mediated transmission (3, 4). Reservoirs for environmentally mediated parasites include domesticated and wild animal host species, vectors like mosquitos and ticks, and abiotic reservoirs like soil and water. Targeting environmental reservoirs could help avert billions of infections and millions of deaths caused by infectious diseases. For example, the protozoan parasites that cause malaria are transmitted by infected mosquitoes, and thus vector management methods, such as insecticide-treated bednets or source population reduction, are effective environmental interventions to reduce malaria (5). Improving environmental disease control can also disrupt the vicious cycles of disease and poverty that thwart progress toward sustainably advancing human and ecosystem health (6–9).

However, environmental sources of infection are often overlooked because they are poorly understood, complex, or outside the purview of classic medical interventions, even when environmental transmission contributes substantially to global disease burdens (2, 10, 11). For example, hope for global dracunculiasis elimination has dimmed as recognition that domestic dogs serve as an alternative reservoir host for the adult worms has grown (12–14).With better information on the environmental components of the world's most burdensome infectious diseases – what they are, where they are, how they're changing – opportunities may arise to complement classical medical prevention and treatment with sustainable environmental interventions that control parasites and pathogens in more effective ways (2). And though we often study environmental reservoirs and spillover (i.e., pathogen transmission across host species) in the context of emerging pathogens like SARS-CoV-2 (15–17), the preceding examples illustrate that environmental transmission and sustainable environmental control can also be important for established and neglected diseases (18, 19). Here, we present a database that synthesizes the environmental components of 150 human parasites and pathogens that cause a high burden of disability and loss of life as identified by the World Health Organization (WHO). These WHO-tracked parasites and pathogens include those that cause the big three diseases (tuberculosis, HIV/AIDs, malaria), sexually transmitted infections, diarrheal diseases, childhood-cluster diseases, meningitis, encephalitis, hepatitis, parasitic and vectorborne diseases, intestinal nematode infections, and leprosy.

## Quantifying Environmental Persistence

**Figure 1:**
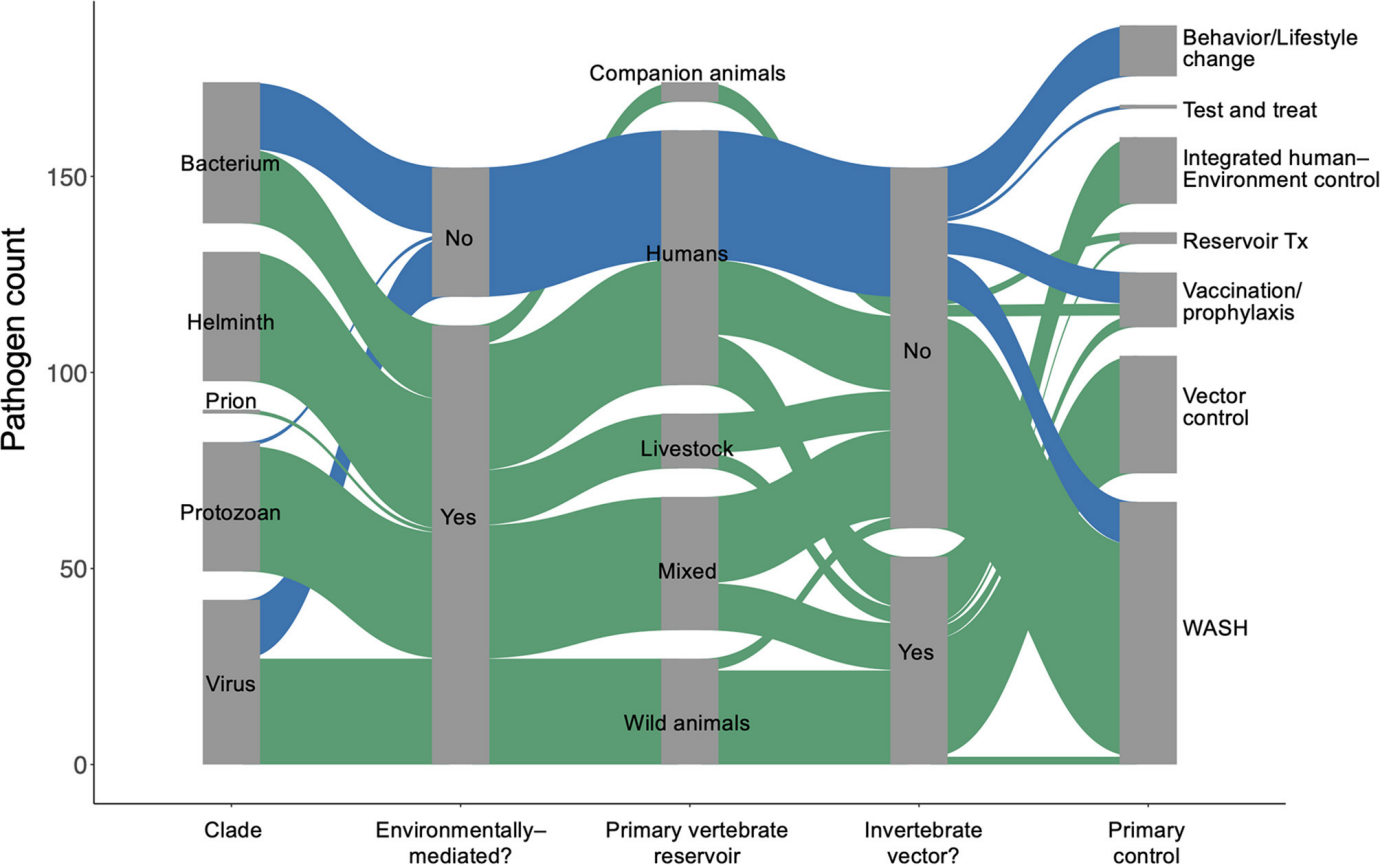
An overview of the 150 most burdensome parasites and pathogens that infect humans.

We used the World Health Organization's Global Health Estimates (GHE) for 2019 (1) to generate a list of 150 parasites and pathogens (see full database in Supplementary Materials) that contribute to significant disability and loss of life, measured in disability-adjusted life years (DALYs) (20). We used a coded list of diseases associated with each “infectious and parasitic disease” category tracked in the GHE (20) to identify the specific species in our list, which included viruses, prions, bacteria, protozoans, and helminths (Figure 1). We then performed a rapid review (21) for each parasite or pathogen, targeting information about (i) the dominant transmission mode, (ii) obligate and incidental vertebrate reservoir hosts, (iii) duration of each life cycle stage in humans and in components of the abiotic or biotic environment, (iv) current global case burdens in humans, and (v) the ‘gold standard' control strategy recommended by global health organizations for each disease (see detailed Methods in Supplementary Materials). Recent global case estimates were surprisingly difficult to find and sometimes varied by source, so we estimated cases using a categorical variable on a logarithmic scale (i.e., 0–100 cases; 101–1,000 cases; 1,001–10,000 cases, etc.) to minimize misclassification. The data presented here represents a living database that will need to be updated as our understanding of these pathogens evolves.

This review adds to previous literature describing life history traits of human pathogens by providing a breakdown of how each parasite or pathogen spends time in humans, non-human vertebrate hosts, vectors or invertebrate intermediate hosts, and abiotic reservoirs. Whereas many studies assess the environmental persistence of single emerging and established diseases by describing pathogens' interactions in space—transmission pathways, abiotic environmental reservoirs, and host and vector ranges [e.g., (15, 17, 22, 23)]—we also include the time that infectious organisms spend, on average, in environmental stages of their life cycles. After compiling these data, we describe the relationships between environmental persistence and dominant transmission pathways, obligate and relevant vertebrate host ranges, contemporary estimates of the global cases of disease, and standard strategies for control and prevention. We explain emergent patterns in this review, and we provide the full database in the supplement as a resource for scientists and practitioners interested in integrated human–environment disease management.

## Pathogen Duration in the Environment Varies Within and Among Transmission Pathways

**Figure 2:**
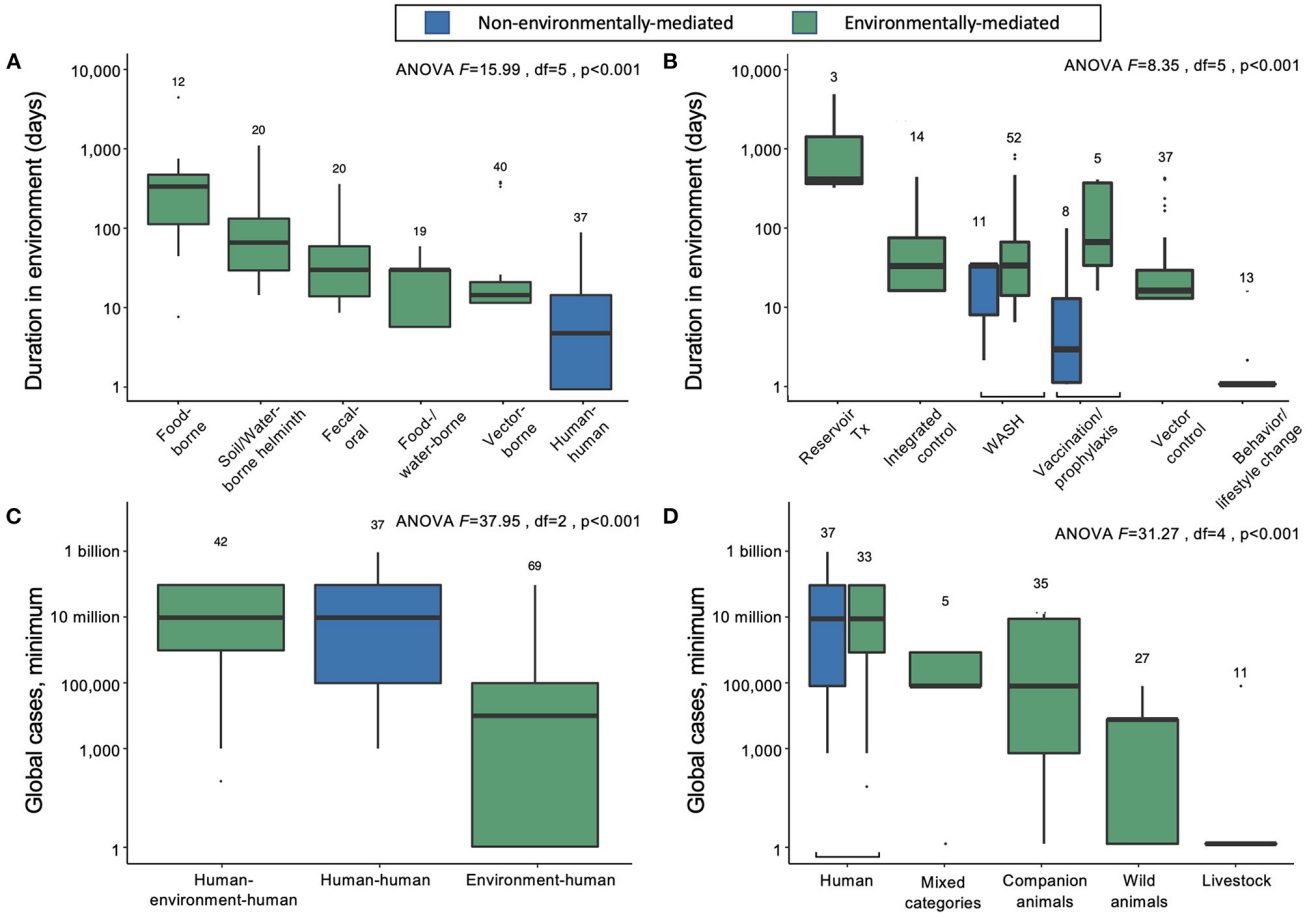
**(A)** Duration of infectious stages outside primary vertebrate hosts (but including vertebrates as obligate intermediate hosts) according to primary transmission strategy, excluding normal flora/opportunistic pathogens and directly zoonotic pathogens, which were data-limited, and sapronoses, which persist indefinitely in abiotic reservoirs. **(B)** Duration of infectious stages outside primary vertebrate hosts according to standard strategies for disease prevention and control, excluding sapronoses. **(C)** The minimum estimated global cases according to obligate transmission pathways, categorized as direct human-to-human transmission (e.g., STDs), human-to-human transmission with obligate vertebrate stages (e.g., soil-transmitted helminths), and environment-to-human transmission (e.g., rabies virus). **(D)** The minimum estimated global cases by obligate vertebrate host ranges; mixed category includes some combination of livestock, poultry, domestic animals, wild animals (including birds), or humans.

Approximately 75% of the world's 150 most burdensome infectious diseases are environmentally mediated, while 10% (primarily STDs) have no potential to survive outside of a human or vertebrate host for more than 1 day. The remaining 15% are considered directly transmitted (human-to-human) diseases with only very brief persistence outside a human host. However, even among pathogens considered to be “directly transmitted” (i.e., acquired via direct human-to-human transmission), there is considerable variability in environmental persistence (Figure 2A). For example, most tuberculosis transmission occurs via respiratory droplets that spend seconds to min in the air during close contacts between infectious and susceptible individuals. But in ideal conditions, *Mycobacterium tuberculosis* can survive and remain virulent for weeks to months outside of a human host. Overall, environmental persistence times for pathogens with potential to survive outside a host vary from just a few minutes to many years.

On average, the duration spent in the environment varied with transmission mode. Parasites and pathogens with primarily direct human-to-human transmission pathways (i.e., via fomites or respiratory droplets, or sexual transmission) have the lowest average persistence in the environment outside human or obligate vertebrate hosts (mean = 4.7 days, 95% CI = 1.5–12.0) (Figure 2A). Vector-borne diseases also tend to have relatively short environmental persistence (mean = 6.2 days, 95% CI = 1.5–19.7) due to the shorter lifespans of invertebrate vectors like mosquitoes and flies. For example, adult female *Anopheles* mosquitoes, which transmit malaria, rarely live longer than 1 to 2 weeks in nature, and will be infectious for only a few days after the parasite's extrinsic incubation period is complete (24). Similarly, parasites and pathogens spread through consumption of contaminated food or water have short environmental persistence (mean = 7.2 days, 95% CI = 2.3–23.4), because the free-living environmental stages are usually short lived (e.g., bacteria like *Salmonella*). In contrast, infections resulting from direct consumption of animals (i.e., trophic transmission) have the longest potential duration in the environment (mean = 171.5 days, 95% CI = 61.8–473.0), because vertebrate hosts and the helminths that are passed from animals to people via trophic transmission are relatively long lived (Figure 2A). While there are individual parasites and pathogens that deviate from these averages, the general trends suggest that the effectiveness of interventions that target infectious stages within humans vs. infectious stages within the environment will depend on transmission modes.

## Standard Control and Prevention Strategies Vary By Pathogen Duration in the Environment

For environmentally mediated diseases, the most common strategies for prevention and control are, in descending order: clean water, sanitation, and hygiene (WASH); vector control; and integrated human–environment control (Figure 1B). In addition to those common control strategies, vaccination or pre-exposure prophylaxis is used for six viruses, including rabies and vector borne encephalitis. Furthermore, direct treatment of reservoir vertebrate hosts is used for three infectious diseases found in long-lived mammals and for which humans play no major role in maintaining the pathogen's life cycle: Louping ill virus, *Echinococcus granulosus*, and *Echinococcus multilocularis*. The only control strategy encountered in the database that was not applied as a typical method to control environmentally mediated diseases was behavioral or lifestyle changes (e.g., safe sex or exposure avoidance).

For directly transmitted infectious diseases, WASH (e.g., hand-washing) and behavior or lifestyle change are the key strategies for prevention and control; the latter strategy largely addresses STDs, while the former addresses directly transmitted diseases with relatively longer environmental persistence (estimated marginal mean = 16.1 days; 95% CI = 5.7–42.4), including Human adenovirus A through G, *Bordetella parapertussis, Streptococcus pyogenes* and *S. agalactiae*, and *Staphylococcus aureus*. Vaccination or pre-exposure prophylaxis are also common control and prevention strategies for many directly transmitted diseases, including HIV/AIDS, childhood cluster diseases, Hepatitis B virus, and several bacterial species causing meningitis. As expected, most of these “gold standard” control strategies do not target environmental reservoirs, but environmental control is still useful for some directly transmitted infectious diseases.

Though most parasites and pathogens spend some time in the environment, only 14% (21/150) of the parasites and pathogens considered here had integrated human and environmental control as the gold standard (i.e., combination of medical intervention and environmental control). This included some infectious agents that cause malaria, yellow fever, schistosomiasis, and soil-transmitted helminths (STH), for which medical intervention (prophylaxis, MDA, or vaccine) and environmental control (vector control or WASH) are simultaneously pursued. However, even within these pathogen groups, there was variability; for the malaria, schistosome, and STH parasites that circulate primarily in non-human vertebrates and cause incidental or dead-end infections in humans, medical interventions would cure individual humans but not affect pathogen transmission. Therefore, rather than integrated approaches, environmental control (vector control or WASH) alone was considered the gold standard for those pathogens.

## Environmentally Mediated Infections Cause a Substantial Burden of Disease

Directly transmitted diseases (human–human) and environmentally mediated pathogens that use humans as the main reservoir hosts (human–environment–human) cause the same average minimum number of infections worldwide (Figure 2C). In contrast, pathogens that predominantly circulate in the abiotic or biotic environment (environment–human) cause lower minimum global cases, on average (Figure 2C). Overall, the logarithm of the estimated minimum number of global cases is positively correlated with the duration of infection in humans (linear mixed effects model, estimate for logarithm of duration in humans: 1.11, SE = 0.18, df = 140, *p* < 0.001), rather than total duration in the environment (estimate for logarithm of duration of pathogens outside a vertebrate host in the environment: = −0.16, SE = 0.30, df = 138 *p* = 0.6). However, despite causing fewer minimum global cases per pathogen, the 113 out of 150 infectious diseases that are environmentally mediated cause an average of more than 16.8 million minimum global cases—a substantial burden of disease.

We can further divide the environmentally mediated diseases into those that are (1) maintained exclusively in human hosts (including pathogens with complex life cycles that require human infection to be maintained); (2) maintained in companion animals (i.e., dogs and cats), livestock, or wild animals; or (3) some combination of 1 and 2 (“mixed categories” in Figure 2D). In doing so, it again becomes clear that directly transmitted and environmentally mediated pathogens maintained exclusively in humans cause, on average, a higher burden of disease than pathogens maintained in animal populations. However, many ‘mixed' host pathogens also cause a substantial number of infections worldwide. These include several diarrheal diseases caused by bacterial, viral, and protozoan pathogens (e.g., *Salmonella enterica, E. coli, Campylobacter* spp., *Giardia lamblia*, Rotavirus spp.), as well as mixed-host complex life cycle parasites like Japanese encephalitis virus, *Leishmania donovani, Echinococcus granulosus*, and *Schistosoma mansoni*.

Among parasites and pathogens that use non-human animals as their main reservoir hosts, pathogens that are primarily maintained in companion animals (*n* = 5) cause, on average, the highest number of infections. However, there are relatively few diseases caused by those types of pathogens: hookworm diseases, caused by *Ancylostoma braziliense* and *A. caninum*, and diarrheal diseases, caused by *Cryptosporidium canis* and *C. felis*, each cause 100,000 to 1 million or more cases annually, while Rotavirus I is reported to cause diarrhea in <100 people annually. Pathogens that are primarily maintained in wild animals (*n* = 27) or livestock (*n* = 11) are more common, but these cause a lower number of human infections, on average, with substantial variation among infectious diseases (wild animal estimated marginal mean = 449 minimum cases, 95% CI = 3–7212; livestock estimated marginal mean = 2.30 minimum cases, 95% CI = 1–62). The exception to this trend among pathogens transmitted via livestock is bovine tuberculosis, which caused an estimated 140,000 cases globally in 2019 (25).

## Discussion

By tabulating how long pathogens spend in the environment and where they spend that time, this synthesis reveals important patterns among 150 burdensome human parasites and pathogens, as well as surprising insights for particular infectious agents. We found that parasites and pathogens with similar transmission pathways (e.g., sexual transmission versus food-borne transmission) have similar environmental durations (e.g., short vs. long) and similar control methods (e.g., behavioral interventions vs. sanitation and hygiene). At the same time, environmental duration can vary substantially for individual pathogens. For example, some directly transmitted pathogens that usually spend almost no time in the environment are capable of acting as environmentally mediated pathogens (e.g., Trachoma caused by direct contact *Chlamydia trachomatis* transmission vs. fly vector borne transmission), a potentially underappreciated phenomenon in many epidemiological studies (26). These environmental persistence data can be especially important when predicting infectious disease dynamics; for example, model-based predictions are often highly sensitive to environmental traits like mosquito life spans, so parameterizing these models with accurate estimates is critical for improving quantitative predictions (27–31). Therefore, we hope that this synthetic review and the associated database serve as a resource for understanding both common patterns among pathogens and important variability and uncertainty in environmental persistence, thereby improving systems-based approaches for understanding and controlling environmentally mediated diseases that infect humans.

We found that most of the 150 most burdensome human pathogens spend some time in the environment outside of the human host. This was not the case for a few diseases that cause the highest human burdens (e.g., HIV/AIDS). But some other diseases that cause more than 1 million DALYs globally do have important abiotic or biotic reservoirs, such as schistosomiasis, Chagas disease, and other neglected tropical diseases. Furthermore, although the most burdensome pathogens primarily use humans now, many originated from spillover events from non-human animals; in other words, although pathogens that are primarily maintained in non-human hosts cause relatively low human disease burdens now, spillover into human hosts can lead to sustained human-to-human transmission and subsequent mass mortality or disability (32), as the HIV/AIDs and COVID-19 pandemics have illustrated. Therefore, by increasing awareness of environmental reservoirs and focusing on reducing environmental transmission, we might simultaneously control the most burdensome endemic/neglected infectious diseases (2, 11) and reduce the risk of emergence of new infectious diseases in human populations (33, 34).

The environmental persistence data synthesized here were often difficult to find, even though we were reviewing the most burdensome and thus often best studied infectious diseases that infect humans. In some cases, the data simply did not exist and needed to be inferred from closely related species. In other cases, the data were spread across multiple sources with variation in their estimates. This emphasizes how many research gaps likely exist for lesser-known diseases, including those that are currently emerging or re-emerging. These data gaps can be a major barrier to successful control; for example, uncertainty about the life cycle and transmission of *Mycobacterium ulcerans* has hampered efforts to control the debilitating Buruli ulcer disease (35–37). Therefore, there is still high value in detailed life cycle research that determines where and how parasites and pathogens spend their time, even though such life cycle studies are becoming less common in the parasitological literature (38, 39).

Planetary Health research increasingly documents how human health is strongly tied to the environment via infectious diseases. Since most of the 150 most burdensome human infectious diseases reviewed here spend time in the environment outside of the human host, it is clear that environmental change might affect many diseases in many places [e.g., 3, 34]. For example, environmental changes could exacerbate vicious cycles of endemic disease, poverty, and environmental degradation (6–9). Environmental changes could also lead to new interactions between humans and environments or shifting host, vector, or pathogen distributions that lead to the emergence or re-emergence of novel pathogens in human populations [e.g., (3, 34, 40). To understand, predict, and control these current and future risks to human health, we must continue working toward understanding how and where infectious agents spend their time outside of human hosts.

## Author Contributions

GD, SS, SH, and IJ conceived of the database idea. IJ, SH, CL, NN, LK, and JB collected and analyzed the data. All authors contributed to drafting and editing the manuscript. All authors contributed to the article and approved the submitted version.

## Funding

This research was conducted by the Ecological Levers for Health expert working group supported by the Science for Nature and People Partnership (SNAPP), a collaboration of The Nature Conservancy, the Wildlife Conservation Society, and the National Center for Ecological Analysis and Synthesis (NCEAS) at the University of California, Santa Barbara. IJ was supported by a National Science Foundation Graduate Research Fellowship (#1656518). NN was supported by the Stanford Data Science Scholars program, the Stanford Center for Computational, Evolutionary and Human Genomics (CEHG) Predoctoral Fellowship, and the Philanthropic Educational Organization (P.E.O.) Scholar Award, International Chapter of the P.E.O. Sisterhood. AL was supported by a James and Nancy Kelso Fellowship through the Stanford Interdisciplinary Graduate Fellowship program. AM was supported by a National Science Foundation Postdoctoral Research Fellowship in Biology (#1611767), and by the National Science Foundation and Fogarty International Center (DEB-2032276, DEB-2011147). GD was supported by NSF DEB 2011179 and the Bill and Melinda Gates Foundation (OPP1114050).

## Conflict of Interest

The authors declare that the research was conducted in the absence of any commercial or financial relationships that could be construed as a potential conflict of interest.

## Publisher's Note

All claims expressed in this article are solely those of the authors and do not necessarily represent those of their affiliated organizations, or those of the publisher, the editors and the reviewers. Any product that may be evaluated in this article, or claim that may be made by its manufacturer, is not guaranteed or endorsed by the publisher.
