# Supplementary material for: Environmental Persistence of the World's Most Burdensome Infectious and Parasitic Diseases
Source: Front Public Health. 2022 Jul 8;10:892366. doi: 10.3389/fpubh.2022.892366 (PMC9305703; doi:10.3389/fpubh.2022.892366)
Supplement: Supplementary file 2 [file Data_Sheet_1.pdf]

Supplementary Material

1 Supplementary Methods

Database construction

Since 1990, the World Health Organization and collaborating organizations have attempted to generate comparative data on the global burden of diseases and injuries and update this dataset over time. Here we used the recently updated Global Burden of Disease (GBD) estimates for 2000–2019 as a foundation to identify major categories of infectious and parasitic diseases of global health importance, for which disability-adjusted life years (DALYs) and years of life lost due to disability (YLDs) have been calculated (20). We used ICD-10 codes provided for "infectious and parasitic" Global Health Estimates (GHE) to identify causative agents of disease to species, but excluded diseases categorized in "other" causes and any ICD-10 codes associated with unspecified causes of disease (20). This resulted in 150 individual species, which we refer to as 150 "infectious diseases" throughout. For each infectious disease, we characterized the dominant transmission strategy, identified obligate and relevant non-human host ranges, and estimated the amount of time each infectious organism spends in human, animal, and environmental reservoirs during the course of its life cycle (distinguishing incubation periods from duration of infectiousness).

Transmission strategies included: human-to-human; fecal–oral; food-borne; soil-borne; water-borne; vector-borne; zoonotic (i.e. direct contact between humans and wild or domestic vertebrates); and sapronotic (saprophages, free-living organisms that consume dead plant and animal biomass, infect humans opportunistically (22)). Among helminths, only schistosomes use water-borne transmission as a primary transmission strategy, so we merged water-borne helminths with soil-borne helminths for visualizations and descriptive statistics. Diseases acquired through fecal–oral transmission are often characterized as human-to-human transmission, but we distinguished the fecal–oral strategy from direct transmission via respiratory droplets and close, intimate contact. For diseases that can be transmitted via multiple routes, we included the strategy assumed to cause the greatest number of infections, but we also provide a "secondary" transmission route in the database. For example, *Vibrio cholerae* is sometimes considered a sapronotic or water-borne disease (22), but because many large outbreaks are associated with fecal-oral transmission within households or communities (41,42), we classify it as a fecal-oral pathogen, with water-borne transmission as a secondary strategy.

For diseases with non-human vertebrate hosts, we classified the animal vertebrate hosts as: (i) obligate hosts, where humans are incidental or dead-end hosts; or (ii) relevant alternative hosts, where vertebrates maintain parasite and pathogen populations independently from humans, and can therefore contribute to disease in certain contexts at the animal–human interface and may compromise long-term control. The primary vertebrate host was classified as: humans only; wild animals (including birds); companion animals (i.e., dogs or cats); livestock; or, mixed, where humans and non-human vertebrates can both maintain pathogen populations. Vertebrate host ranges (i.e., all known host species used by the pathogen), provided descriptively, are therefore likely to change as new data emerges for diseases that are currently less well understood.

Duration was characterized as time (in days) that a single infectious organism spends in the following contexts: in vertebrate hosts (including humans); as free-living stages in abiotic environments; or in obligate vectors or intermediate hosts. We attempted to distinguish the duration of incubation periods

from infectious periods, but this information is lacking for many pathogens. For many parasites and pathogens, human (or vertebrate) infectious periods can be cyclical or interspersed with dormant stages; for consistency, we estimated duration of infectiousness as the average duration of infection, even though this may overestimate the actual period spent shedding enough infectious material to cause infection in other hosts. For these and other reasons, the total time an infectious organism spends in various stages of its life cycle does not represent its generation time. Moreover, disease duration can be highly variable in humans and environmental reservoirs depending on host body condition, temperature, humidity, and other physical and biological factors. For this reason, we include a description of duration according to data found in literature sources in the supplemental database.

We also collected data on the contemporary, or most recently available, total number of cases each infectious organism causes in humans in a given year. Because source estimates of global cases are highly variable, sometimes outdated, and often difficult to find, we reduced the chances of assigning an incorrect estimate of global cases by assigning cases to logarithmic categories (i.e., 0–100; 101–1,000; 1,001–10,000, 10,000–100,000; 100,000–1 million; >1 million; >1 billion). Rare and neglected diseases that lack centralized reporting systems and diseases that do not typically require medical treatment are likely underestimated.

Finally, we included data on the ‘gold standard’ control strategy recommended by global health organizations for each disease or closely related diseases. Control strategies include (i) behavior or lifestyle change (e.g., safe sex); (ii) vaccination or pre-exposure prophylaxis; (iii) water, sanitation, and hygiene (WASH) or safe food preparation; (iv) vector control; (v) reservoir host treatment (e.g., animal vaccination); and (vi) integrated human and environmental control (e.g., combinations of multiple strategies such as mass drug administration and environmental control). We note that some “gold standard” prevention strategies, like pre-exposure prophylactics (PrEP) for HIV, are not accessible or affordable to those who may need them.

Between 2017 and 2020 we conducted extensive literature searches to collect data on the ecology of rare diseases and to estimate duration of time spent in human and environmental reservoirs (incubation and infectious periods). Where possible, data were acquired from peer-reviewed scientific literature, the World Health Organization, the U.S. Centers for Disease Control and Prevention, and health communications resources provided by other academic, national, and global health institutions (i.e., (43)). Peer-reviewed sources used included laboratory experiments, modeling exercises, and reviews.

The life cycles, host ranges, environmental persistence, and control strategies of many diseases are still being investigated, and data presented here is likely to change as more knowledge is generated. Therefore, we encourage users to consider this database as a first step in synthesizing data on the life cycle, host diversity and environmental persistence of parasites and pathogens causing a substantial burden of human infectious disease, and to further investigate specific diseases of interest.

Descriptive statistics

We used linear mixed effects ANOVAs to describe (i) how duration of infectious stages outside obligate vertebrate hosts varies with primary transmission strategy; (ii) how duration of infectious stages outside vertebrate obligate hosts varies with gold standard control and prevention practices; (iii) how global cases (minimum value of logarithmic range) varies with transmission pathways; and (iv) how global cases vary with obligate vertebrate host range. Both outcome variables (duration of infectious stages and global cases) were scaled using a natural logarithm. We included a random

effect term for the 11 major disease categories assigned in the WHO GBD database (Tuberculosis, STDs excluding HIV, HIV/AIDS, Diarrheal diseases, Childhood-cluster diseases, Meningitis, Encephalitis, Hepatitis, Parasitic and vector diseases, Intestinal nematode infections, Leprosy), excluding 'Other infectious diseases'. All analyses were performed in the statistical computing software, R, version 1.3 (44) and models were built using the 'lme4' package (45). Estimated marginal means for categorical predictors were estimated using the 'emmeans' package (46).

References for Supplementary Methods:

20. WHO. WHO methods and data sources for global burden of disease estimates 2000-2019. Geneva: World Health Organization; 2020 Dec. Report No.: WHO/DDI/DNA/GHE/2020.3.
22. Kuris AM, Lafferty KD, Sokolow SH. Sapronosis: a distinctive type of infectious agent. Trends in Parasitology. 2014 Aug;30(8):386–93.
41. Clemens JD, Nair GB, Ahmed T, Qadri F, Holmgren J. Cholera. The Lancet. 2017 Sep 23;390(10101):1539–49.
42. Meszaros VA, Miller-Dickson MD, Junior FB-A, Almagro-Moreno S, Ogbunugafor CB. Direct transmission via households informs models of disease and intervention dynamics in cholera. PLOS ONE. 2020 Mar 12;15(3):e0229837.
43. Spickler AR. Technical Factsheets [Internet]. The Center for Food Security and Public Health; 2020 [cited 2020 Jul 1]. Available from: http://www.cfsph.iastate.edu/DiseaseInfo/factsheets.php
44. R Core Team. R: A language and environment for statistical computing [Internet]. Vienna, Austria: R Foundation for Statistical Computing; 2020. Available from: https://www.R-project.org/
45. Bates D, Mächler M, Bolker B, Walker S. Fitting Linear Mixed-Effects Models Using lme4. J Stat Soft [Internet]. 2015 [cited 2020 Jun 16];67(1). Available from: http://www.jstatsoft.org/v67/i01/
46. Lenth R. emmeans: Estimated Marginal Means, aka Least-Squares Means [Internet]. 2020. Available from: https://CRAN.R-project.org/package=emmeans

2 Supplementary Data

See the attached database.

3 References for Supplementary Data

1. WHO. *Global Tuberculosis Report 2019*. http://www.who.int/tb/publications/global_report/en/ (2019).
2. Blower, S. M. & Chou, T. Modeling the emergence of the 'hot zones': tuberculosis and the amplification dynamics of drug resistance. *Nat. Med.* **10**, 1111–1116 (2004).
3. Dye, C. & Espinal, M. A. Will tuberculosis become resistant to all antibiotics? *Proc. R. Soc. Lond. B Biol. Sci.* **268**, 45–52 (2001).
4. Walther, B. A. & Ewald, P. W. Pathogen survival in the external environment and the evolution of virulence. *Biol. Rev.* **79**, 849–869 (2004).
5. Montali, R. J., Mikota, S. K. & Cheng, L. I. Mycobacterium tuberculosis in zoo and wildlife species. **20**, 291 (2001).
6. Spickler, A. *Zoonotic Tuberculosis in Mammals, including Bovine and Caprine Tuberculosis*. http://www.cfsph.iastate.edu/Factsheets/pdfs/bovine_tuberculosis.pdf (2019).

7. Boston University. Agent Information Sheets Database | Research Support. *Boston University Research Support* https://www.bu.edu/researchsupport/safety/rohp/agent-information-sheets/.
8. CDC. Diseases & Conditions Database. https://www.cdc.gov/DiseasesConditions/ (2021).
9. WHO. *Zoonotic Tuberculosis*. https://www.who.int/tb/zoonoticTB.pdf (2017).
10. Cosivi, O. *et al.* Zoonotic tuberculosis due to Mycobacterium bovis in developing countries. *Emerg. Infect. Dis.* **4**, 59–70 (1998).
11. Anguelov, R., Garba, S. M. & Usaini, S. Backward bifurcation analysis of epidemiological model with partial immunity. *Comput. Math. Appl.* **68**, 931–940 (2014).
12. National Research Council. *Livestock Disease Eradication: Evaluation of the Cooperative State-Federal Bovine Tuberculosis Eradication Program*. (The National Academies Press, 1994). doi:10.17226/9144.
13. WHO. *Report on global sexually transmitted infection surveillance 2018*. http://www.who.int/reproductivehealth/publications/stis-surveillance-2018/en/ (2018).
14. Rowley, J. *et al.* Chlamydia, gonorrhoea, trichomoniasis and syphilis: global prevalence and incidence estimates, 2016. *Bull. World Health Organ.* **97**, 548-562P (2019).
15. WHO. Fact Sheets Database. *World Health Orginization* https://www.who.int/news-room/fact-sheets (n.d.).
16. CDC. Sexually Transmitted Diseases Database. *CDC-Centers for Disease Control and Prevention* https://www.cdc.gov/std/default.htm (2020).
17. Newman, L. *et al.* Global Estimates of Syphilis in Pregnancy and Associated Adverse Outcomes: Analysis of Multinational Antenatal Surveillance Data. *PLOS Med.* **10**, e1001396 (2013).
18. Grassly, N. C., Fraser, C. & Garnett, G. P. Host immunity and synchronized epidemics of syphilis across the United States. *Nature* **433**, 417–421 (2005).
19. Public Health Agency of Canada. Pathogen Safety Data Sheets Database. *aem* https://www.canada.ca/en/public-health/services/laboratory-biosafety-biosecurity/pathogen-safety-data-sheets-risk-assessment.html (2021).
20. Radolf, J. D. Treponema. in *Medical Microbiology* (ed. Baron, S.) (University of Texas Medical Branch at Galveston, 1996).
21. WHO. *WHO guidelines for the treatment of Treponema pallidum (syphilis)*. http://www.who.int/reproductivehealth/publications/rtis/syphilis-treatment-guidelines/en/ (2016).
22. Chuma, I. S. *et al.* Widespread Treponema pallidum Infection in Nonhuman Primates, Tanzania. *Emerg. Infect. Dis.* **24**, 1002–1009 (2018).
23. Tien, V., Punjabi, C. & Holubar, M. K. Antimicrobial resistance in sexually transmitted infections. *J. Travel Med.* **27**, (2020).
24. Rank, R. G. & Yeruva, L. Hidden in Plain Sight: Chlamydial Gastrointestinal Infection and Its Relevance to Persistence in Human Genital Infection. *Infect. Immun.* **82**, 1362–1371 (2014).
25. Althaus, C. L., Choisy, M. & Alizon, S. *How sex acts scale with the number of sex partners: evidence from Chlamydia trachomatis data and implications for control*. https://peerj.com/preprints/940 (2015) doi:10.7287/peerj.preprints.940v2.
26. Sandoz, K. M. & Rockey, D. D. Antibiotic resistance in Chlamydiae. *Future Microbiol.* **5**, 1427–1442 (2010).
27. Newman, L. *et al.* Global Estimates of the Prevalence and Incidence of Four Curable Sexually Transmitted Infections in 2012 Based on Systematic Review and Global Reporting. *PloS One* **10**, e0143304 (2015).

28. Geisler, W. M. Duration of untreated, uncomplicated Chlamydia trachomatis genital infection and factors associated with chlamydia resolution: a review of human studies. *J. Infect. Dis.* **201 Suppl 2**, S104-113 (2010).
29. Jolly, A. M. & Wylie, J. L. Gonorrhoea and chlamydia core groups and sexual networks in Manitoba. *Sex. Transm. Infect.* **78**, i145–i151 (2002).
30. Herold, A. H. *et al.* Seasonality of Chlamydia Trachomatis Genital Infections in University Women. *J. Am. Coll. Health* **42**, 117–120 (1993).
31. Spickler, A. *Zoonotic Chlamydiae Maintained in Mammals*. http://www.cfsph.iastate.edu/Factsheets/pdfs/chlamydiosis.pdf (2017).
32. CDC. 2015 STD Treatment Guidelines. https://www.cdc.gov/std/tg2015/default.htm (2015).
33. Schroeder, B., Tetlow, P., Sanfilippo, J. S. & Hertweck, S. P. Is there a seasonal variation in gonorrhea and chlamydia in adolescents? *J. Pediatr. Adolesc. Gynecol.* **14**, 25–27 (2001).
34. Edwards, R., Kim, S. & van den Driessche, P. A multigroup model for a heterosexually transmitted disease. *Math. Biosci.* **224**, 87–94 (2010).
35. Free Safety Data Sheet Index. *MSDSonline* https://www.msdsonline.com/resources/sds-resources/free-safety-data-sheet-index/ (2018).
36. Kissinger, P. Trichomonas vaginalis: a review of epidemiologic, clinical and treatment issues. *BMC Infect. Dis.* **15**, (2015).
37. Kirkcaldy, R. D. *et al.* Trichomonas vaginalis Antimicrobial Drug Resistance in 6 US Cities, STD Surveillance Network, 2009–2010. *Emerg. Infect. Dis.* **18**, 939–943 (2012).
38. James, C. *et al.* Herpes simplex virus: global infection prevalence and incidence estimates, 2016. *Bull. World Health Organ.* **98**, 315–329 (2020).
39. WHO. Globally, an estimated two-thirds of the population under 50 are infected with herpes simplex virus type 1. *World Health Orginization* https://www.who.int/news/item/28-10-2015-globally-an-estimated-two-thirds-of-the-population-under-50-are-infected-with-herpes-simplex-virus-type-1 (2015).
40. Jiang, Y.-C., Feng, H., Lin, Y.-C. & Guo, X.-R. New strategies against drug resistance to herpes simplex virus. *Int. J. Oral Sci.* **8**, 1–6 (2016).
41. Looker, K. J. *et al.* Global Estimates of Prevalent and Incident Herpes Simplex Virus Type 2 Infections in 2012. *PLoS ONE* **10**, e114989 (2015).
42. Newton, E. A. & Kuder, J. M. A model of the transmission and control of genital herpes. *Sex. Transm. Dis.* **27**, 363–370 (2000).
43. Chayavichitsilp, P., Buckwalter, J. V., Krakowski, A. C. & Friedlander, S. F. Herpes Simplex. *Pediatr. Rev.* **30**, 119–130 (2009).
44. González-Beiras, C., Marks, M., Chen, C. Y., Roberts, S. & Mitjà, O. Epidemiology of Haemophilus ducreyi Infections. *Emerg. Infect. Dis.* **22**, 1–8 (2016).
45. Steen, R. Eradicating chancroid. *Bull. World Health Organ.* **79**, 818–826 (2001).
46. Al-Tawfiq, J. A. & Spinola, S. M. Haemophilus ducreyi: clinical disease and pathogenesis. *Curr. Opin. Infect. Dis.* **15**, 43–47 (2002).
47. Houinei, W. *et al.* Haemophilus ducreyi DNA is detectable on the skin of asymptomatic children, flies and fomites in villages of Papua New Guinea. *PLoS Negl. Trop. Dis.* **11**, e0004958 (2017).
48. Richens, J. The diagnosis and treatment of donovanosis (granuloma inguinale). *Genitourin. Med.* **67**, 441–452 (1991).
49. Martel, C. de, Plummer, M., Vignat, J. & Franceschi, S. Worldwide burden of cancer attributable to HPV by site, country and HPV type. *Int. J. Cancer* **141**, 664–670 (2017).

50. The HPV TEST. *QIAGEN* https://herqiagen.com/hpv/ (2019).
51. Rosa, L. Papillomavirus. *Global Water Pathogen Project* https://www.waterpathogens.org/book/papillomavirus (2016).
52. Juckett, G. & Hartman-Adams, H. Human Papillomavirus: Clinical Manifestations and Prevention. *Am. Fam. Physician* **82**, 1209–1214 (2010).
53. Williams, B. G. *et al.* Epidemiological Trends for HIV in Southern Africa: Implications for Reaching the Elimination Targets. *Curr. HIV/AIDS Rep.* **12**, 196–206 (2015).
54. Holtgrave, D. R. On the Epidemiologic and Economic Importance of the National AIDS Strategy for the United States. *JAIDS J. Acquir. Immune Defic. Syndr.* **55**, 139–142 (2010).
55. Spach, D. H. & Budak, J. Z. HIV-2 Infection. *National HIV Curriculum* https://www.hiv.uw.edu/go/key-populations/hiv-2/core-concept/all (2020).
56. *ABC of HIV and AIDS*. (WIley-Blackwell [u.a.], 2012).
57. Global HIV and AIDS statistics. *Avert* https://www.avert.org/global-hiv-and-aids-statistics (2015).
58. Salomon, J. A. *et al.* Disability weights for the Global Burden of Disease 2013 study. *Lancet Glob. Health* **3**, e712–e723 (2015).
59. HIV.gov. The Global HIV/AIDS Epidemic. *HIV.gov* https://www.hiv.gov/hiv-basics/overview/data-and-trends/global-statistics (2019).
60. Core Concepts - HIV-2 Infection - Key Populations. *National HIV Curriculum* https://www.hiv.uw.edu/go/key-populations/hiv-2/core-concept/all (2020).
61. Kotloff, K. L. *et al.* Global burden of Shigella infections: implications for vaccine development and implementation of control strategies. *Bull. World Health Organ.* **77**, 651–666 (1999).
62. Khalil, I. A. *et al.* Morbidity and mortality due to shigella and enterotoxigenic Escherichia coli diarrhoea: the Global Burden of Disease Study 1990–2016. *Lancet Infect. Dis.* **18**, 1229–1240 (2018).
63. Joh, R. I. *et al.* Dynamics of shigellosis epidemics: estimating individual-level transmission and reporting rates from national epidemiologic data sets. *Am. J. Epidemiol.* **178**, 1319–1326 (2013).
64. Jennison, A. V. & Verma, N. K. Shigella flexneri infection: pathogenesis and vaccine development. *FEMS Microbiol. Rev.* **28**, 43–58 (2004).
65. Troeger, C. *et al.* Estimates of the global, regional, and national morbidity, mortality, and aetiologies of diarrhoea in 195 countries: a systematic analysis for the Global Burden of Disease Study 2016. *Lancet Infect. Dis.* **18**, 1211–1228 (2018).
66. WHO. *Guidelines for the control of shigellosis, including epidemics due to Shigella dysenteriae type 1*. 64 https://www.who.int/maternal_child_adolescent/documents/9241592330/en/ (2005).
67. University of Adleide. Shigella Flexneri. *School of Biological Sciences*.
68. Kramer, A., Schwebke, I. & Kampf, G. How long do nosocomial pathogens persist on inanimate surfaces? A systematic review. *BMC Infect. Dis.* **6**, (2006).
69. Sansonetti, P. J. III. Shigellosis: from symptoms to molecular pathogenesis. *Am. J. Physiol.-Gastrointest. Liver Physiol.* **280**, G319–G323 (2001).
70. Farag, T. H. *et al.* Housefly Population Density Correlates with Shigellosis among Children in Mirzapur, Bangladesh: A Time Series Analysis. *PLoS Negl. Trop. Dis.* **7**, e2280 (2013).
71. Thompson, C. N., Duy, P. T. & Baker, S. The Rising Dominance of Shigella sonnei: An Intercontinental Shift in the Etiology of Bacillary Dysentery. *PLoS Negl. Trop. Dis.* **9**, e0003708 (2015).
72. Filliol-Toutain, I. *et al.* Global Distribution of Shigella sonnei Clones. *Emerg. Infect. Dis.* **17**, 1910–1912 (2011).

73. Bacteria and Viruses. *FoodSafety.gov* https://www.foodsafety.gov/food-poisoning/bacteria-and-viruses (2019).
74. Pitzer, V. E. *et al.* Predicting the Impact of Vaccination on the Transmission Dynamics of Typhoid in South Asia: A Mathematical Modeling Study. *PLoS Negl. Trop. Dis.* **8**, e2642 (2014).
75. Douesnard-Malo, F. & Daigle, F. Increased Persistence of Salmonella enterica Serovar Typhi in the Presence of Acanthamoeba castellanii ▿ . *Appl. Environ. Microbiol.* **77**, 7640–7646 (2011).
76. Salmonellosis. *Department of Health & Human Services* https://www2.health.vic.gov.au:443/public-health/infectious-diseases/disease-information-advice/salmonellosis.
77. Stanaway, J. D. *et al.* The global burden of typhoid and paratyphoid fevers: a systematic analysis for the Global Burden of Disease Study 2017. *Lancet Infect. Dis.* **19**, 369–381 (2019).
78. Saad, N. J. *et al.* Seasonal dynamics of typhoid and paratyphoid fever. *Sci. Rep.* **8**, 6870 (2018).
79. Crump, J. A., Luby, S. P. & Mintz, E. D. The global burden of typhoid fever. *Bull. World Health Organ.* **82**, 346–353 (2004).
80. Majowicz, S. E. *et al.* The Global Burden of Nontyphoidal Salmonella Gastroenteritis. *Clin. Infect. Dis.* **50**, 882–889 (2010).
81. Kingsley, R. A. & Bäumler, A. J. Host adaptation and the emergence of infectious disease: the Salmonella paradigm. *Mol. Microbiol.* **36**, 1006–1014 (2000).
82. Jajere, S. M. A review of Salmonella enterica with particular focus on the pathogenicity and virulence factors, host specificity and antimicrobial resistance including multidrug resistance. *Vet. World* **12**, 504–521 (2019).
83. Pava-Ripoll, M. *et al.* Ingested Salmonella enterica, Cronobacter sakazakii, Escherichia coli O157:H7, and Listeria monocytogenes: transmission dynamics from adult house flies to their eggs and first filial (F1) generation adults. *BMC Microbiol.* **15**, 150 (2015).
84. Jijón, S., Wetzel, A. & LeJeune, J. SALMONELLA ENTERICA ISOLATED FROM WILDLIFE AT TWO OHIO REHABILITATION CENTERS. *J. Zoo Wildl. Med.* **38**, 409–413 (2007).
85. French, E., Rodriguez-Palacios, A. & LeJeune, J. T. Enteric bacterial pathogens with zoonotic potential isolated from farm-raised deer. *Foodborne Pathog. Dis.* **7**, 1031–1037 (2010).
86. Chiari, M. *et al.* Isolation and identification of Salmonella spp. from red foxes (Vulpes vulpes) and badgers (Meles meles) in northern Italy. *Acta Vet. Scand.* **56**, (2014).
87. Stoddard, R. A., DeLong, R. L., Byrne, B. A., Jang, S. & Gulland, F. M. D. Prevalence and characterization of Salmonella spp. among marine animals in the Channel Islands, California. *Dis. Aquat. Organ.* **81**, 5–11 (2008).
88. Crump, J. A. & Wain, J. Salmonella. in *International Encyclopedia of Public Health (Second Edition)* (ed. Quah, S. R.) 425–433 (Academic Press, 2017). doi:10.1016/B978-0-12-803678-5.00394-5.
89. Ali, M., Nelson, A. R., Lopez, A. L. & Sack, D. A. Updated Global Burden of Cholera in Endemic Countries. *PLoS Negl. Trop. Dis.* **9**, (2015).
90. Diarrhea - Symptoms and causes. *Mayo Clinic* https://www.mayoclinic.org/diseases-conditions/diarrhea/symptoms-causes/syc-20352241.
91. Dangbé, E., Irépran, D., Perasso, A. & Békollé, D. Mathematical modelling and numerical simulations of the influence of hygiene and seasons on the spread of cholera. *Math. Biosci.* **296**, 60–70 (2018).
92. Jubair, M., Jr, J. G. M. & Ali, A. Survival of Vibrio cholerae in Nutrient-Poor Environments Is Associated with a Novel “Persister” Phenotype. *PLOS ONE* **7**, e45187 (2012).

93. Kenyon, J. E., Piexoto, D. R., Austin, B. & Gillies, D. C. Seasonal variations of Vibrio cholerae (non-O1) isolated from California coastal waters. *Appl. Environ. Microbiol.* **47**, 1243–1245 (1984).
94. Bouma, M. J. & Pascual, M. Seasonal and interannual cycles of endemic cholera in Bengal 1891–1940 in relation to climate and geography. in *The Ecology and Etiology of Newly Emerging Marine Diseases* (ed. Porter, J. W.) 147–156 (Springer Netherlands, 2001). doi:10.1007/978-94-017-3284-0_13.
95. Levine, M. M. *et al.* Duration of infection-derived immunity to cholera. *J. Infect. Dis.* **143**, 818–820 (1981).
96. Van der Henst, C. *et al.* Molecular insights into Vibrio cholerae's intra-amoebal host-pathogen interactions. *Nat. Commun.* **9**, (2018).
97. Freeman, J. T., Anderson, D. J. & Sexton, D. J. Seasonal peaks in Escherichia coli infections: possible explanations and implications. *Clin. Microbiol. Infect. Off. Publ. Eur. Soc. Clin. Microbiol. Infect. Dis.* **15**, 951–953 (2009).
98. Chen, S. *et al.* Basic Reproduction Number and Transmission Dynamics of Common Serogroups of Enterohemorrhagic Escherichia coli. *Appl. Environ. Microbiol.* **82**, 5612–5620 (2016).
99. Gopee, N. V., Adesiyun, A. A. & Caesar, K. A longitudinal study of Escherichia coli strains isolated from captive mammals, birds, and reptiles in Trinidad. *J. Zoo Wildl. Med. Off. Publ. Am. Assoc. Zoo Vet.* **31**, 353–360 (2000).
100. Levine, M. M. *et al.* Immunity to Enterotoxigenic Escherichia coli. *Infect. Immun.* **23**, 729–736 (1979).
101. Collignon, P. Resistant Escherichia coli— We Are What We Eat. *Clin. Infect. Dis.* **49**, 202–204 (2009).
102. Spickler, A. *Enterohemorrhagic Escherichia coli and Other E. coli Causing Hemolytic Uremic Syndrome*. https://www.cfsph.iastate.edu/diseaseinfo/factsheets/ (2016).
103. *WHO estimates of the global burden of foodborne diseases*. (World Health Organization, 2015).
104. Kaneko, K. & Hashimoto, N. Occurrence of Yersinia enterocolitica in wild animals. *Appl. Environ. Microbiol.* **41**, 635–638 (1981).
105. Fredriksson-Ahomaa, M. *et al.* Yersiniosis in zoo marmosets (Callitrix jacchuss) caused by Yersinia enterocolitica. *Vet. Microbiol.* **121**, 363–367 (2007).
106. Rahman, A., Bonny, T. S., Stonsaovapak, S. & Ananchaipattana, C. Yersinia enterocolitica: Epidemiological Studies and Outbreaks. *J. Pathog.* **2011**, 239391 (2011).
107. Sabina, Y., Rahman, A., Ray, R. C. & Montet, D. Yersinia enterocolitica: Mode of Transmission, Molecular Insights of Virulence, and Pathogenesis of Infection. *J. Pathog.* **2011**, (2011).
108. Food and Drug Administration. *Bad Bug Book: Foodborne Pathogenic Microorganisms and Natural Toxins - Second Edition*. (FDA, 2012).
109. YERSINIA ENTEROCOLITICA: Pathogen Safety Sheet. *University of Texas Austin* https://www.uta.edu/campus-ops/ehs/biological/docs/PSDS/YERSINIA%20ENTEROCOLITICA.pdf.
110. Tambur, Z. Ispitivanje osetljivosti na antibiotike Campylobacter jejuni i C. coli izolovanih iz ljudi. *Vojnosanit. Pregl.* **66**, 49–53 (2009).
111. Louis, V. R. *et al.* Temperature-Driven Campylobacter Seasonality in England and Wales. *Appl. Environ. Microbiol.* **71**, 85–92 (2005).
112. Kaakoush, N. O., Castaño-Rodríguez, N., Mitchell, H. M. & Man, S. M. Global Epidemiology of Campylobacter Infection. *Clin. Microbiol. Rev.* **28**, 687–720 (2015).
113. Tam, C. Campylobacter coli—an important foodborne pathogen. *J. Infect.* **47**, 28–32 (2003).

114. Havelaar, A. H. *et al.* Immunity to Campylobacter: its role in risk assessment and epidemiology. *Crit. Rev. Microbiol.* **35**, 1–22 (2009).
115. Taema, M. M. *et al.* Retrospective Study of Campylobacter Infection in a Zoological Collection | Applied and Environmental Microbiology. *Appl Environ Microbiol* **74**, 1332–1338 (2008).
116. Horrocks, S. M., Anderson, R. C., Nisbet, D. J. & Ricke, S. C. Incidence and ecology of Campylobacter jejuni and coli in animals. *Anaerobe* **15**, 18–25 (2009).
117. Jones, K. Campylobacters in water, sewage and the environment. *J. Appl. Microbiol.* **90**, 68S-79S (2001).
118. Spickler, A. *Zoonotic Campylobacteriosis*. https://www.cfsph.iastate.edu/diseaseinfo/factsheets/ (2013).
119. Laughlin, M. E., Chatham-Stephens, K. & Geissler, A. L. Chapter 4: Travel-Related Infectious Diseases | Campylobacteriosis. in *CDC Health Information for International Travel 2020 - The Yellow Book* vol. 23 (CDC, 2019).
120. WHO. *The global view of campylobacteriosis: Report of an Expert Consultation*. https://www.who.int/foodsafety/publications/campylobacteriosis/en/ (2013).
121. Masila, N. M., Ross, K. E., Gardner, M. G. & Whiley, H. Zoonotic and Public Health Implications of Campylobacter Species and Squamates (Lizards, Snakes and Amphisbaenians). *Pathogens* **9**, (2020).
122. Prince Milton, A. A. *et al.* Prevalence of Campylobacter jejuni and Campylobacter coli in captive wildlife species of India. *Iran. J. Vet. Res.* **18**, 177–182 (2017).
123. Mendz, G. L., Petersen, R., Quinlivan, J. A. & Kaakoush, N. O. Potential involvement of Campylobacter curvus and Haemophilus parainfluenzae in preterm birth. *Case Rep.* **2014**, bcr2014205282–bcr2014205282 (2014).
124. Wetsch, N. M. *et al.* Campylobacter curvus-Associated Hepatic Abscesses: a Case Report. *J. Clin. Microbiol.* **44**, 1909–1911 (2006).
125. Abbott, S. L. *et al.* Description of Campylobacter curvus and C. curvus-Like Strains Associated with Sporadic Episodes of Bloody Gastroenteritis and Brainerd’s Diarrhea. *J. Clin. Microbiol.* **43**, 585–588 (2005).
126. Liu, F., Ma, R., Wang, Y. & Zhang, L. The Clinical Importance of Campylobacter concisus and Other Human Hosted Campylobacter Species. *Front. Cell. Infect. Microbiol.* **8**, (2018).
127. Wilkinson, D. A. *et al.* Updating the genomic taxonomy and epidemiology of Campylobacter hyointestinalis. *Sci. Rep.* **8**, (2018).
128. Waldenström, J. *et al.* Prevalence of Campylobacter jejuni, Campylobacter lari, and Campylobacter coli in Different Ecological Guilds and Taxa of Migrating Birds. *Appl. Environ. Microbiol.* **68**, 5911–5917 (2002).
129. Miller, W. G. *et al.* Comparative genomics of the Campylobacter lari group. *Genome Biol. Evol.* **6**, 3252–3266 (2014).
130. Platts-Mills, J. A., Kosek, M. N., Allos, B. M. & Blaser, M. J. Campylobacter species - Infectious Disease and Antimicrobial Agents. *Antimicrobe* http://www.antimicrobe.org/b91.asp (2010).
131. Swedish University of Agricultural Science. Campylobacter lari. *VetBact* http://www.vetbact.org/index.php?artid=144 (2020).
132. García-Peña, F. J. *et al.* Isolation and Characterization of Campylobacter spp. from Antarctic Fur Seals (Arctocephalus gazella) at Deception Island, Antarctica. *Appl. Environ. Microbiol.* **76**, 6013–6016 (2010).
133. Bronowski, C., James, C. E. & Winstanley, C. Role of environmental survival in transmission of Campylobacter jejuni. *FEMS Microbiol. Lett.* **356**, 8–19 (2014).

134. Bourke, B., Chan, V. L. & Sherman, P. Campylobacter upsaliensis: Waiting in the Wings. *Clin. Microbiol. Rev.* **11**, 440–449 (1998).
135. Karama, M. *et al.* Occurrence and Antimicrobial Resistance Profiles of Campylobacter jejuni, Campylobacter coli, and Campylobacter upsaliensis in Beef Cattle on Cow-Calf Operations in South Africa. *Foodborne Pathog. Dis.* **17**, 440–446 (2020).
136. Sinulingga, T. S., Aziz, S. A., Bitrus, A. A., Zunita, Z. & Abu, J. Occurrence of Campylobacter species from broiler chickens and chicken meat in Malaysia. *Trop. Anim. Health Prod.* **52**, 151–157 (2020).
137. Wagenaar, J. A. *et al.* Campylobacter fetus Infections in Humans: Exposure and Disease. *Clin. Infect. Dis.* **58**, 1579–1586 (2014).
138. Allos, B. M., Iovine, N. M. & Blaser, M. J. Campylobacter jejuni and Related Species. in *Mandell, Douglas, and Bennett's Principles and Practice of Infectious Diseases (Eighth Edition)* (eds. Bennett, J. E., Dolin, R. & Blaser, M. J.) vol. 2 2485-2493.e4 (W.B. Saunders, 2015).
139. Tee, W., Luppino, M. & Rambaldo, S. Bacteremia due to Campylobacter sputorum Biovar sputorum. *Clin. Infect. Dis. Off. Publ. Infect. Dis. Soc. Am.* **27**, 1544–1545 (1998).
140. Miller, W. G., Yee, E., Chapman, M. H. & Bono, J. L. Comparative Genomics of All Three Campylobacter sputorum Biovars and a Novel Cattle-Associated C. sputorum Clade. *Genome Biol. Evol.* **9**, 1513–1518 (2017).
141. Solomon, K. The host immune response to Clostridium difficile infection. *Ther. Adv. Infect. Dis.* **1**, 19–35 (2013).
142. Gilca, R., Fortin, É., Frenette, C., Longtin, Y. & Gourdeau, M. Seasonal Variations in Clostridium difficile Infections Are Associated with Influenza and Respiratory Syncytial Virus Activity Independently of Antibiotic Prescriptions: a Time Series Analysis in Québec, Canada. *Antimicrob. Agents Chemother.* **56**, 639–646 (2012).
143. CDC. Healthcare-associated Infections Database. https://www.cdc.gov/hai/index.html (2021).
144. Lanzas, C., Dubberke, E. R., Lu, Z., Reske, K. A. & Gröhn, Y. T. Epidemiological Model for *Clostridium difficile* Transmission in Healthcare Settings. *Infect. Control Hosp. Epidemiol.* **32**, 553–561 (2011).
145. Balsells, E. *et al.* Infection prevention and control of Clostridium difficile: a global review of guidelines, strategies, and recommendations. *J. Glob. Health* **6**, (2016).
146. Keel, M. K. & Songer, J. G. The Comparative Pathology of Clostridium difficile-associated Disease. *Vet. Pathol.* **43**, 225–240 (2006).
147. Moono, P. *et al.* Clostridium difficile Infection in Production Animals and Avian Species: A Review | Foodborne Pathogens and Disease. *Foodborne Pathog. Dis.* **13**, (2016).
148. Weese, J. S. Clostridium (Clostridioides) difficile in animals. *J. Vet. Diagn. Invest.* **32**, 213–221 (2020).
149. Torgerson, P. R. *et al.* World Health Organization Estimates of the Global and Regional Disease Burden of 11 Foodborne Parasitic Diseases, 2010: A Data Synthesis. *PLOS Med.* **12**, e1001920 (2015).
150. Asgharpour, A., Gilchrist, C., Baba, D., Hamano, S. & Houpt, E. Resistance to intestinal Entamoeba histolytica infection is conferred by innate immunity and Gr-1+ cells. *Infect. Immun.* **73**, 4522–4529 (2005).
151. Nagata, N. *et al.* Risk Factors for Intestinal Invasive Amebiasis in Japan, 2003–2009. *Emerg. Infect. Dis.* **18**, 717–724 (2012).

152. Penuliar, G. M., Furukawa, A., Sato, D. & Nozaki, T. Mechanism of trifluoromethionine resistance in Entamoeba histolytica. *J. Antimicrob. Chemother.* **66**, 2045–2052 (2011).
153. CDC. DPDx Database. https://www.cdc.gov/dpdx/az.html (2019).
154. Patton, S. Overview of Amebiasis - Digestive System. *Merck Veterinary Manual* https://www.merckvetmanual.com/digestive-system/amebiasis/overview-of-amebiasis (2013).
155. Harvard Medical School. Gastrointestinal Amebiasis. *Harvard Health Publishing* https://www.health.harvard.edu/a_to_z/gastrointestinal-amebiasis-a-to-z (2019).
156. Arizona Department of Health Services. Common Enteric Pathogens. https://www.azdhs.gov/documents/preparedness/epidemiology-disease-control/infectious-diseases-training/2017/presentations/handout2-common-enteric-pathogens-table-web.pdf.
157. Arean, V. M. & Koppisch, E. Balantidiasis; a review and report of cases. *Am. J. Pathol.* **32**, 1089–1115 (1956).
158. Schuster, F. L. & Ramirez-Avila, L. Current World Status of Balantidium coli. *Clin. Microbiol. Rev.* **21**, 626–638 (2008).
159. Flanagan, P. A. Giardia--diagnosis, clinical course and epidemiology. A review. *Epidemiol. Infect.* **109**, 1–22 (1992).
160. Spickler, A. *Giardiasis*. https://www.cfsph.iastate.edu/Factsheets/pdfs/giardiasis.pdf (2012).
161. Pumipuntu, N. & Piratae, S. Cryptosporidiosis: A zoonotic disease concern. *Vet. World* **11**, 681–686 (2018).
162. Medema, G. J., Bahar, M. & Schets, F. M. Survival of Cryptosporidium parvum, Escherichia coli, faecal enterococci and Clostridium perfringens in river water: influence of temperature and autochthonous microorganisms. *Water Sci. Technol.* **35**, 249–252 (1997).
163. Fayer, R. *et al.* Cryptosporidium parvum infection in bovine neonates: dynamic clinical, parasitic and immunologic patterns. *Int. J. Parasitol.* **28**, 49–56 (1998).
164. Perch, M. *et al.* Seven years' experience with Cryptosporidium parvum in Guinea-Bissau, West Africa. *Ann. Trop. Paediatr.* **21**, 313–318 (2001).
165. O'Handley, R. M. *et al.* Duration of naturally acquired giardiosis and cryptosporidiosis in dairy calves and their association with diarrhea. *J. Am. Vet. Med. Assoc.* **214**, 391–396 (1999).
166. Semenza, J. C. & Nichols, G. Cryptosporidiosis surveillance and water-borne outbreaks in Europe. *Eurosurveillance* **12**, 13–14 (2007).
167. Spickler, A. *Cryptosporidiosis*. https://www.cfsph.iastate.edu/Factsheets/pdfs/cryptosporidiosis.pdf (2018).
168. The Australian Society for Parasitology Inc. Cryptosporidium. http://parasite.org.au/para-site/text/cryptosporidium-text.html (2019).
169. CAB International. Invasive Species Compendium. *CABI* https://www.cabi.org/isc/ (n.d.).
170. Xiao, L. *et al.* Genetic Diversity of Cryptosporidium spp. in Captive Reptiles. *Appl. Environ. Microbiol.* **70**, 891–899 (2004).
171. Zahedi, A., Paparini, A., Jian, F., Robertson, I. & Ryan, U. Public health significance of zoonotic Cryptosporidium species in wildlife: Critical insights into better drinking water management. *Int. J. Parasitol. Parasites Wildl.* **5**, 88–109 (2016).
172. Leitch, G. J. & He, Q. Cryptosporidiosis-an overview. *J. Biomed. Res.* **25**, 1–16 (2011).
173. Sheoran, A., Wiffin, A., Widmer, G., Singh, P. & Tzipori, S. Infection With Cryptosporidium hominis Provides Incomplete Protection of the Host Against Cryptosporidium parvum. *J. Infect. Dis.* **205**, 1019–1023 (2012).
174. Widmer, G., Köster, P. & Carmena, D. Cryptosporidium hominis infections in non-human animal species: revisiting the concept of host specificity. *Int. J. Parasitol.* **50**, (2020).

175. Mayo Clinic. Cryptosporidium Infection - Symptoms & causes. https://www.mayoclinic.org/diseases-conditions/cryptosporidium/symptoms-causes/syc-20351870 (2019).
176. King, B. J., Keegan, A. R., Monis, P. T. & Saint, C. P. Environmental Temperature Controls Cryptosporidium Oocyst Metabolic Rate and Associated Retention of Infectivity. *Appl. Environ. Microbiol.* **71**, 3848–3857 (2005).
177. Koompapong, K. & Sukthana, Y. Seasonal variation and potential sources of Cryptosporidium contamination in surface waters of Chao Phraya River and Bang Pu Nature Reserve pier, Thailand. *Southeast Asian J. Trop. Med. Public Health* **43**, 832–840 (2012).
178. MN State. Causes and Symptoms of Cryptosporidiosis. https://www.health.state.mn.us/diseases/cryptosporidiosis/basics.html (2018).
179. Chappell, C. L. *et al.* Cryptosporidium meleagridis: infectivity in healthy adult volunteers. *Am. J. Trop. Med. Hyg.* **85**, 238–242 (2011).
180. Cacciò, S. M. & Putignani, L. Epidemiology of Human Cryptosporidiosis. in *Cryptosporidium: parasite and disease* (eds. Cacciò, S. M. & Widmer, G.) 43–79 (Springer Vienna, 2014). doi:10.1007/978-3-7091-1562-6_2.
181. Huang, D. B., Chappell, C. & Okhuysen, P. C. Cryptosporidiosis in children. *Semin. Pediatr. Infect. Dis.* **15**, 253–259 (2004).
182. Olson, M. E., Goh, J., Phillips, M., Guselle, N. & McAllister, T. A. Giardia Cyst and Cryptosporidium Oocyst Survival in Water, Soil, and Cattle Feces. *J. Environ. Qual.* **28**, 1991–1996 (1999).
183. Ryan, U., Fayer, R. & Xiao, L. Cryptosporidium species in humans and animals: current understanding and research needs. *Parasitology* **141**, 1667–1685 (2014).
184. Dubey, J. P. & Almeria, S. Cystoisospora belli infections in humans: the past 100 years. *Parasitology* **146**, 1490–1527 (2019).
185. Lindsay, D. & Weiss, L. Isospora belli. http://www.antimicrobe.org/b04rev.asp (2010).
186. Weiss, O. S., Ludington, J. G., Ward, H. D. & Weiss, L. M. Cystoisosporiasis. in *Encyclopedia of AIDS* (eds. Hope, T. J., Stevenson, M. & Richman, D.) 1–4 (Springer New York, 2015). doi:10.1007/978-1-4614-9610-6_450-1.
187. Rodriguez-Morales, A. J. & Castañeda-Hernández, D. M. Protozoa: Cystoisospora belli (Syn. Isospora belli). in *Encyclopedia of Food Safety* 45–48 (Elsevier, 2014). doi:10.1016/B978-0-12-378612-8.00136-0.
188. Nozawa, T. & Matsubayashi, H. Experimental Infection of Isospora Hominis in Man 1. *Am. J. Trop. Med. Hyg.* **s1-28**, 633–637 (1948).
189. Faust, E. C., Giraldo, L. E., Caicedo, G. & Bonfante, R. Human Isosporosis in the Western Hemisphere. *Am. J. Trop. Med. Hyg.* **10**, 343–349 (1961).
190. Humphrey, A. A. ISOSPORA HOMINIS INFECTION IN MAN. *J. Am. Med. Assoc.* **130**, 143 (1946).
191. Connal, A. Observations on the pathogenicity of Isospora hominis, Rivolta, emend. Dobell, based on a second case of human coccidiosis in Nigeria; with remarks on the significance of Charcot-Leyden crystals in the f1ces. *Trans. R. Soc. Trop. Med. Hyg.* **16**, 223–245 (1922).
192. Fayer, R. Sarcocystis spp. in Human Infections. *Clin. Microbiol. Rev.* **17**, 894–902 (2004).
193. Fayer, R., Esposito, D. H. & Dubey, J. P. Human Infections with Sarcocystis Species. *Clin. Microbiol. Rev.* **28**, 295–311 (2015).
194. McKenna, P. B. & Charleston, W. A. G. The survival of Sarcocystis gigantea sporocysts following exposure to various chemical and physical agents. *Vet. Parasitol.* **45**, 1–16 (1992).

195. Spickler, A. *Sarcocystosis*. http://www.cfsph.iastate.edu/Factsheets/pdfs/sarcocystosis.pdf (2020).
196. Roberts, L. S., Janovy, J. & Nadler, S. *Foundations of Parasitology*. (McGraw Hill, 2000).
197. Ortega, Y. R. & Sanchez, R. Update on Cyclospora cayetanensis, a food-borne and waterborne parasite. *Clin. Microbiol. Rev.* **23**, 218–234 (2010).
198. Almeria, S., Cinar, H. N. & Dubey, J. P. Cyclospora cayetanensis and Cyclosporiasis: An Update. *Microorganisms* **7**, 317 (2019).
199. Pitzer, V. E. *et al.* Modeling rotavirus strain dynamics in developed countries to understand the potential impact of vaccination on genotype distributions. *Proc. Natl. Acad. Sci.* **108**, 19353–19358 (2011).
200. Ansari, S. A., Springthorpe, V. S. & Sattar, S. A. Survival and vehicular spread of human rotaviruses: possible relation to seasonality of outbreaks. *Rev. Infect. Dis.* **13**, 448–461 (1991).
201. Rao, V. C., Seidel, K. M., Goyal, S. M., Metcalf, T. G. & Melnick, J. L. Isolation of enteroviruses from water, suspended solids, and sediments from Galveston Bay: survival of poliovirus and rotavirus adsorbed to sediments. *Appl. Environ. Microbiol.* **48**, 404–409 (1984).
202. Malik, Y. S. *et al.* Evolving Rotaviruses, Interspecies Transmission and Zoonoses. *Open Virol. J.* **14**, (2020).
203. Ghosh, S. & Kobayashi, N. Exotic rotaviruses in animals and rotaviruses in exotic animals. *VirusDisease* **25**, 158–172 (2014).
204. Coria-Galindo, E. *et al.* ROTAVIRUS INFECTIONS IN GALAPAGOS SEA LIONS. *J. Wildl. Dis.* **45**, 722–728 (2009).
205. de Blasio, B. F., Kasymbekova, K. & Flem, E. Dynamic model of rotavirus transmission and the impact of rotavirus vaccination in Kyrgyzstan. *Vaccine* **28**, 7923–7932 (2010).
206. Sanekata, T., Ahmed, M. U., Kader, A., Taniguchi, K. & Kobayashi, N. Human group B rotavirus infections cause severe diarrhea in children and adults in Bangladesh. *J. Clin. Microbiol.* **41**, 2187–2190 (2003).
207. Kim, Y., Chang, K. O., Straw, B. & Saif, L. J. Characterization of group C rotaviruses associated with diarrhea outbreaks in feeder pigs. *J. Clin. Microbiol.* **37**, 1484–1488 (1999).
208. Rahman, M. *et al.* Detection and Characterization of Human Group C Rotaviruses in Bangladesh. *J. Clin. Microbiol.* **43**, 4460–4465 (2005).
209. Pitzer, V. E. *et al.* Demographic Variability, Vaccination, and the Spatiotemporal Dynamics of Rotavirus Epidemics. *Science* **325**, 290–294 (2009).
210. Parashar, U. D., Hummelman, E. G., Bresee, J. S., Miller, M. A. & Glass, R. I. Global illness and deaths caused by rotavirus disease in children. *Emerg. Infect. Dis.* **9**, 565–572 (2003).
211. Deol, P. *et al.* Avian Group D Rotaviruses: Structure, Epidemiology, Diagnosis, and Perspectives on Future Research Challenges. *Pathogens* **6**, (2017).
212. Kattoor, J. J. *et al.* Development of VP6 Gene Specific Reverse Transcription(RT)-PCR Assay for Detectionof Avian Group D Rotavirus in Diarrheic Chickens. *J. Vet. Sci. Med. Diagn.* **03**, (2014).
213. Wakuda, M. *et al.* Porcine Rotavirus Closely Related to Novel Group of Human Rotaviruses. *Emerg. Infect. Dis.* **17**, 1491–1493 (2011).
214. Phan, T. G., Leutenegger, C. M., Chan, R. & Delwart, E. Rotavirus I in feces of a cat with diarrhea. *Virus Genes* **53**, 487–490 (2017).
215. Ogorzaly, L., Bertrand, I., Paris, M., Maul, A. & Gantzer, C. Occurrence, Survival, and Persistence of Human Adenoviruses and F-Specific RNA Phages in Raw Groundwater. *Appl. Environ. Microbiol.* **76**, 8019–8025 (2010).

216. Crabtree, K. D., Gerba, C. P., Rose, J. B. & Haas, C. N. Waterborne adenovirus: a risk assessment. *Water Sci. Technol.* **35**, 1–6 (1997).
217. Jiang, S. C. Human Adenoviruses in Water: Occurrence and Health Implications: A Critical Review. *Environ. Sci. Technol.* **40**, 7132–7140 (2006).
218. Rzeżutka, A. & Cook, N. Survival of human enteric viruses in the environment and food. *FEMS Microbiol. Rev.* **28**, 441–453 (2004).
219. Lessler, J. *et al.* Incubation periods of acute respiratory viral infections: a systematic review. *Lancet Infect. Dis.* **9**, 291–300 (2009).
220. Harrach, B., Tarján, Z. L. & Benkő, M. Adenoviruses across the animal kingdom: a walk in the zoo - Harrach - 2019 - FEBS Letters - Wiley Online Library. *FEBS Lett.* **593**, 3660–3673 (2019).
221. CDC *et al.* Human Adenovirus Surveillance — United States, 2003–2016. *MMWR Morb. Mortal. Wkly. Rep.* **66**, 1039–1042 (2017).
222. Ghebremedhin, B. Human adenovirus: Viral pathogen with increasing importance. *Eur. J. Microbiol. Immunol.* **4**, 26–33 (2014).
223. Pond, K. & WHO. *Water recreation and disease: plausibility of associated infections, acute effects, sequelae and mortality*. (IWA Publisher, 2005).
224. Pauly, M. *et al.* High prevalence and diversity of species D adenoviruses (HAdV-D) in human populations of four Sub-Saharan countries. *Virol. J.* **11**, 25 (2014).
225. Patel, M. M., Hall, A. J., Vinjé, J. & Parashar, U. D. Noroviruses: A comprehensive review. *J. Clin. Virol.* **44**, 1–8 (2009).
226. Villabruna, N., Koopmans, M. P. G. & de Graaf, M. Animals as Reservoir for Human Norovirus. *Viruses* **11**, (2019).
227. Kilgore, P. E., Salim, A. M., Zervos, M. J. & Schmitt, H.-J. Pertussis: Microbiology, Disease, Treatment, and Prevention. *Clin. Microbiol. Rev.* **29**, 449–486 (2016).
228. König, C.-H. W. von. Pertussis diagnostics: overview and impact of immunization. *Expert Rev. Vaccines* **13**, 1167–1174 (2014).
229. Althouse, B. M. & Scarpino, S. V. Asymptomatic transmission and the resurgence of Bordetella pertussis. *BMC Med.* **13**, (2015).
230. Metcalf, C. J. E., Bjørnstad, O. N., Grenfell, B. T. & Andreasen, V. Seasonality and comparative dynamics of six childhood infections in pre-vaccination Copenhagen. *Proc. R. Soc. B Biol. Sci.* **276**, 4111–4118 (2009).
231. Jackson, D. W. & Rohani, P. Perplexities of pertussis: recent global epidemiological trends and their potential causes. *Epidemiol. Infect.* **142**, 672–684 (2014).
232. Yeung, K. H. T., Duclos, P., Nelson, E. A. S. & Hutubessy, R. C. W. An update of the global burden of pertussis in children younger than 5 years: a modelling study. *Lancet Infect. Dis.* **17**, 974–980 (2017).
233. WHO. Health Topics Database. *World Health Orginization* https://www.who.int/health-topics (2021).
234. Cherry, J. D. The Present and Future Control of Pertussis. *Clin. Infect. Dis.* **51**, 663–667 (2010).
235. Washington State Department of Health. Whooping Cough (pertussis) in Washington. *Washington State Department of Health* https://www.doh.wa.gov/YouandYourFamily/IllnessandDisease/WhoopingCough.
236. Syed, M. A., Jamil, B. & Bokhari, H. Shattering a myth - Whooping cough susceptible to antibiotics. *Pak. J. Pharm. Sci.* **29**, 985–990 (2016).

237. Mastrantonio, P. *et al.* Bordetella parapertussis Infection in Children: Epidemiology, Clinical Symptoms, and Molecular Characteristics of Isolates. *J. Clin. Microbiol.* **36**, 999–1002 (1998).
238. Wolfe, D. N., Goebel, E. M., Bjornstad, O. N., Restif, O. & Harvill, E. T. The O Antigen Enables Bordetella parapertussis To Avoid Bordetella pertussis-Induced Immunity. *Infect. Immun.* **75**, 4972–4979 (2007).
239. Bolding. Whooping cough caused by Bordetella parapertussis. *ResearchGate* (2004).
240. Minnesota Dept. of Health. Parapertussis and Holmesii. *Minnesota Dept. of Health* https://www.health.state.mn.us/diseases/pertussis/parapertussis.html (2019).
241. *B. parapertussis Infection: Public Health Recommendations*. 1 https://www.cdph.ca.gov/Programs/CID/DCDC/CDPH%20Document%20Library/Immunization/ParapertussisQuicksheet.pdf (2017).
242. Zakikhany, K. & Efstratiou, A. Diphtheria in Europe: current problems and new challenges. *Future Microbiol.* **7**, 595–607 (2012).
243. Henricson, B. *et al.* Toxigenic Corynebacterium Diphtheriae Associated with an Equine Wound Infection. *J. Vet. Diagn. Invest.* **12**, 253–257 (2000).
244. Barraud, O., Badell, E., Denis, F., Guiso, N. & Ploy, M.-C. Antimicrobial Drug Resistance in Corynebacterium diphtheriae mitis. *Emerg. Infect. Dis.* **17**, 2078–2080 (2011).
245. Clarke, K. E. N. *et al.* Global Epidemiology of Diphtheria, 2000–2017. *Emerg. Infect. Dis.* **25**, (2019).
246. WHO. WHO vaccine-preventable diseases: monitoring system 2021 global summary. https://apps.who.int/immunization_monitoring/globalsummary (2020).
247. Guerra, F. M. *et al.* The basic reproduction number (R0) of measles: a systematic review. *Lancet Infect. Dis.* **17**, e420–e428 (2017).
248. Brook, I. Current concepts in the management of Clostridium tetani infection. *Expert Rev. Anti Infect. Ther.* **6**, 327–336 (2008).
249. Kyu, H. H. *et al.* Mortality from tetanus between 1990 and 2015: findings from the global burden of disease study 2015. *BMC Public Health* **17**, 179 (2017).
250. Behrens, H., Ochmann, S., Dadonaite, B. & Roser, M. Tetanus. *Our World Data* (2019).
251. Popoff, M. R. Tetanus in animals. *J. Vet. Diagn. Invest.* **32**, 184–191 (2020).
252. Vyas, J. M. Tetanus. *MedlinePlus: Medical Encyclopedia* https://medlineplus.gov/ency/article/000615.htm (2019).
253. Wu, H. M. *et al.* Emergence of Ciprofloxacin-Resistant Neisseria meningitidis in North America. *N. Engl. J. Med.* **360**, 886–892 (2009).
254. Gabutti, G., Stefanati, A. & Kuhdari, P. Epidemiology of Neisseria meningitidis infections: case distribution by age and relevance of carriage. *J. Prev. Med. Hyg.* **56**, E116–E120 (2015).
255. Pollard, A. J. & Frasch, C. Development of natural immunity to Neisseria meningitidis. *Vaccine* **19**, 1327–1346 (2001).
256. Mbaeyi, S. A. & McNamara. Chapter 4: Travel-Related Infectious Diseases | Meningococcal Disease. in *CDC Health Information for International Travel 2020 - The Yellow Book* (CDC, 2019).
257. Chuang, S.-Y. Reinfection of Invasive Streptococcus pneumoniae — Analysis of Notifiable Diseases Database in Taiwan. *Taiwan Centers for Disease Control* https://www.cdc.gov.tw/En/EpidemicTheme/Detail/hQ4XhaZAzUmNe2ksY4tjMA?archiveId=6ra7-XxH93GXp7fMmbKQFg (2010).
258. Hébert-Dufresne, L. & Althouse, B. M. Complex dynamics of synergistic coinfections on realistically clustered networks. *Proc. Natl. Acad. Sci.* **112**, 10551–10556 (2015).

259. Chanter, N. Streptococci and enterococci as animal pathogens. *J. Appl. Microbiol.* **83**, 100S-109S (1997).
260. Hoban, D. J., Doern, G. V., Fluit, A. C., Roussel-Delvallez, M. & Jones, R. N. Worldwide prevalence of antimicrobial resistance in Streptococcus pneumoniae, Haemophilus influenzae, and Moraxella catarrhalis in the SENTRY Antimicrobial Surveillance Program, 1997-1999. *Clin. Infect. Dis. Off. Publ. Infect. Dis. Soc. Am.* **32 Suppl 2**, S81-93 (2001).
261. Spickler, A. *Streptococcosis*. http://www.cfsph.iastate.edu/Factsheets/pdfs/streptococcosis.pdf (2005).
262. Charles River Laboratories. Infectious Agent Information | Charles River. *Charles River* https://www.criver.com/products-services/research-models-services/animal-health-surveillance/infectious-agent-information?region=3601 (2011).
263. Langan, G. P., Lohmiller, J. J., Swing, S. P. & Wardrip, C. L. Respiratory Diseases of Rodents and Rabbits. *Vet. Clin. Small Anim. Pract.* **30**, 1309–1335 (2000).
264. WHO. Immunizations, Vaccines, and Biologicals Database. *World Health Orginization* https://www.who.int/teams/immunization-vaccines-and-biologicals/diseases (2021).
265. Sanyahumbi, A. S., Colquhoun, S., Wyber, R. & Carapetis, J. R. Global Disease Burden of Group A Streptococcus. in *Streptococcus pyogenes : Basic Biology to Clinical Manifestations* (eds. Ferretti, J. J., Stevens, D. L. & Fischetti, V. A.) (University of Oklahoma Health Sciences Center, 2016).
266. Savic, D. J. & McShan, W. M. Long-term survival of Streptococcus pyogenes in rich media is pH-dependent. *Microbiology* **158**, 1428–1436 (2012).
267. Wozniak, A., Scioscia, N., Geoffroy, E., Ponce, I. & García, P. Importance of adhesins in the recurrence of pharyngeal infections caused by Streptococcus pyogenes. *J. Med. Microbiol.* **66**, 517–525 (2017).
268. Kanwal, S. & Vaitla, P. Streptococcus Pyogenes. in *StatPearls* (StatPearls Publishing, 2020).
269. Vela, A. I. *et al.* Characterization of Streptococcus pyogenes from Animal Clinical Specimens, Spain. *Emerg. Infect. Dis.* **23**, 2011–2016 (2017).
270. Johri, A. K. *et al.* Group B Streptococcus: global incidence and vaccine development. *Nat. Rev. Microbiol.* **4**, 932–942 (2006).
271. Leelahapongsathon, K., Schukken, Y. H., Pinyopummintr, T. & Suriyasathaporn, W. Comparison of transmission dynamics between Streptococcus uberis and Streptococcus agalactiae intramammary infections. *J. Dairy Sci.* **99**, 1418–1426 (2016).
272. Yang, Q. *et al.* The impact of pH and nutrient stress on the growth and survival of Streptococcus agalactiae. *Antonie Van Leeuwenhoek* **102**, 277–287 (2012).
273. Trotman-Grant, A., Raney, T. & Dien Bard, J. Evaluation of optimal storage temperature, time, and transport medium for detection of group B Streptococcus in StrepB carrot broth. *J. Clin. Microbiol.* **50**, 2446–2449 (2012).
274. Lyhs, U. *et al.* Streptococcus agalactiae Serotype IV in Humans and Cattle, Northern Europe. *Emerg. Infect. Dis.* **22**, (2016).
275. Russell, N. J. *et al.* Maternal Colonization With Group B Streptococcus and Serotype Distribution Worldwide: Systematic Review and Meta-analyses. *Clin. Infect. Dis. Off. Publ. Infect. Dis. Soc. Am.* **65**, S100–S111 (2017).
276. Delannoy, C. M. *et al.* Human Streptococcus agalactiae strains in aquatic mammals and fish. *BMC Microbiol.* **13**, 41 (2013).
277. Tong, S. Y. C., Davis, J. S., Eichenberger, E., Holland, T. L. & Fowler, V. G. Staphylococcus aureus Infections: Epidemiology, Pathophysiology, Clinical Manifestations, and Management. *Clin. Microbiol. Rev.* **28**, 603–661 (2015).

278. Montgomery, C. P. *et al.* Protective Immunity against Recurrent Staphylococcus aureus Skin Infection Requires Antibody and Interleukin-17A. *Infect. Immun.* **82**, 2125–2134 (2014).
279. Levin-Edens, E., Bonilla, N., Meschke, J. S. & Roberts, M. C. Survival of environmental and clinical strains of methicillin-resistant Staphylococcus aureus [MRSA] in marine and fresh waters. *Water Res.* **45**, 5681–5686 (2011).
280. Leekha, S., Diekema, D. J. & Perencevich, E. N. Seasonality of staphylococcal infections. *Clin. Microbiol. Infect. Off. Publ. Eur. Soc. Clin. Microbiol. Infect. Dis.* **18**, 927–933 (2012).
281. Taylor, T. A. & Unakal, C. G. Staphylococcus Aureus. in *StatPearls* (StatPearls Publishing, 2020).
282. Carrillo-Casas, E. M. & Miranda-Morales, R. E. Bovine Mastitis Pathogens: Prevalence and Effects on Somatic Cell Count. *Milk Prod. - --Date Overv. Anim. Nutr. Manag. Health* (2012) doi:10.5772/51032.
283. Prosperi, M. *et al.* Molecular Epidemiology of Community-Associated Methicillin-resistant Staphylococcus aureus in the genomic era: a Cross-Sectional Study. *Sci. Rep.* **3**, (2013).
284. Haag, A. F., Fitzgerald, J. R. & Penadés, J. R. Staphylococcus aureus in Animals. *Microbiol. Spectr.* **7**, (2019).
285. Farrington, C. P., Kanaan, M. N. & Gay, N. J. Estimation of the basic reproduction number for infectious diseases from age-stratified serological survey data. *J. R. Stat. Soc. Ser. C Appl. Stat.* **50**, 251–292 (2001).
286. ECDC. Factsheet about Invasive Haemophilus influenzae disease. *European Centre for Disease Prevention and Control* https://www.ecdc.europa.eu/en/invasive-haemophilus-influenzae-disease/facts.
287. Stanford Children's Health. Haemophilus Influenzae Infections in Children. *Stanford Children's Health* https://www.stanfordchildrens.org/en/topic/default?id=haemophilus-influenzae-infections-90-P02520.
288. WHO. WHO EMRO | Regional Office for the Eastern Mediterranean. *WHO EMRO* http://www.emro.who.int/index.html.
289. WHO. WHO | Haemophilus influenzae type b (Hib). *WHO* https://www.who.int/ith/diseases/HiB/en/ (2014).
290. Campbell, G. *et al.* Estimated global incidence of Japanese encephalitis: a systematic review. *Bull. World Health Organ.* **89**, 766–774 (2011).
291. Khan, S. U. *et al.* Dynamics of Japanese Encephalitis Virus Transmission among Pigs in Northwest Bangladesh and the Potential Impact of Pig Vaccination. *PLoS Negl. Trop. Dis.* **8**, e3166 (2014).
292. Spickler, A. *Japanese Encephalitis*. http://www.cfsph.iastate.edu/Factsheets/pdfs/japanese_encephalitis.pdf (2016).
293. Li, X. *et al.* Lethal Encephalitis in Seals with Japanese Encephalitis Virus Infection, China, 2017. *Emerg. Infect. Dis.* **25**, 1539–1542 (2019).
294. Mansfield, K. L., Hernández-Triana, L. M., Banyard, A. C., Fooks, A. R. & Johnson, N. Japanese encephalitis virus infection, diagnosis and control in domestic animals. *Vet. Microbiol.* **201**, 85–92 (2017).
295. Kumar, K. *et al.* Prevalence and risk factors of Japanese encephalitis virus (JEV) in livestock and companion animal in high-risk areas in Malaysia. *Trop. Anim. Health Prod.* **50**, 741–752 (2018).
296. Ohno, Y. *et al.* Detection of antibodies against Japanese encephalitis virus in raccoons, raccoon dogs and wild boars in Japan. *J. Vet. Med. Sci.* **71**, 1035–1039 (2009).

297. Barba, M., Fairbanks, E. L. & Daly, J. M. Equine viral encephalitis: prevalence, impact, and management strategies. *Vet. Med. Res. Rep.* **10**, 99–110 (2019).
298. Minnesota Dept. of Health. Western Equine Encephalitis Fact Sheet. *Minnesota Dept. of Health* https://www.health.state.mn.us/diseases/weencephalitis/wee.html (2018).
299. Ohio State Department of Health. *Infectious Disease Control Manual: Western Equine Encephalitis Virus Disease*. https://odh.ohio.gov/wps/portal/gov/odh/know-our-programs/infectious-disease-control-manual/section3/section-3-wee (2019).
300. Reisen, W. K., Chiles, R. E., Martinez, V. M., Fang, Y. & Green, E. N. Experimental Infection of California Birds with Western Equine Encephalomyelitis and St. Louis Encephalitis Viruses. *J. Med. Entomol.* **40**, 968–982 (2003).
301. Spickler, A. *Eastern, Western and Venezuelan Equine Encephalomyelitis*. http://www.cfsph.iastate.edu/Factsheets/pdfs/easter_wester_venezuelan_equine_encephalomyelitis.pdf (2017).
302. Boyle, D. B., Dickerman, R. W. & Marshall, I. D. Primary viraemia responses of herons to experimental infection with Murray Valley encephalitis, Kunjin and Japanese encephalitis viruses. *Aust. J. Exp. Biol. Med. Sci.* **61 (Pt 6)**, 655–664 (1983).
303. White, G., Ottendorfer, C., Graham, S. & Unnasch, T. R. Competency of Reptiles and Amphibians for Eastern Equine Encephalitis Virus. *Am. J. Trop. Med. Hyg.* **85**, 421–425 (2011).
304. Chapter 28 - Togaviridae. in *Fenner's Veterinary Virology (Fifth Edition)* (eds. MacLachlan, N. J. & Dubovi, E. J.) 511–524 (Academic Press, 2017). doi:10.1016/B978-0-12-800946-8.00028-3.
305. Brault, A. C. *et al.* Genetic and antigenic diversity among eastern equine encephalitis viruses from North, Central, and South America. *Am. J. Trop. Med. Hyg.* **61**, 579–586 (1999).
306. Armstrong, P. M. & Andreadis, T. G. Eastern Equine Encephalitis Virus in Mosquitoes and Their Role as Bridge Vectors. *Emerg. Infect. Dis.* **16**, (2010).
307. Webster, L. T. IMMUNITY OF MICE FOLLOWING SUBCUTANEOUS VACCINATION WITH ST. LOUIS ENCEPHALITIS VIRUS. *J. Exp. Med.* **68**, 111–124 (1938).
308. Bolling, B. G., Barker, C. M., Moore, C. G., Pape, W. J. & Eisen, L. Seasonal patterns for entomological measures of risk for exposure to Culex vectors and West Nile virus in relation to human disease cases in northeastern Colorado. *J. Med. Entomol.* **46**, 1519–1531 (2009).
309. Buck, C. *et al.* Isolation of St. Louis encephalitis virus from a killer whale. *Clin. Diagn. Virol.* **1**, 109–112 (1993).
310. Day, J. F., Storrs, E. E., Stark, L. M., Lewis, A. L. & Williams, S. Antibodies to St. Louis encephalitis virus in armadillos from southern Florida. *J. Wildl. Dis.* **31**, 10–14 (1995).
311. Rosa, R. *et al.* Isolation of Saint Louis Encephalitis Virus from a Horse with Neurological Disease in Brazil. *PLoS Negl. Trop. Dis.* **7**, (2013).
312. Kopp, A. *et al.* Provenance and Geographic Spread of St. Louis Encephalitis Virus | mBio. *mBio* **4**, e00322-13 (2013).
313. Selvey, L. A. *et al.* The Changing Epidemiology of Murray Valley Encephalitis in Australia: The 2011 Outbreak and a Review of the Literature. *PLoS Negl. Trop. Dis.* **8**, e2656 (2014).
314. Ho, S. H., Speldewinde, P. & Cook, A. A Bayesian Belief Network for Murray Valley encephalitis virus risk assessment in Western Australia. *Int. J. Health Geogr.* **15**, 6 (2016).
315. McLean, D. M. Transmission of Murray Valley Encephalitis Virus by Mosquitoes. *Aust. J. Exp. Biol. Med. Sci.* **31**, 481–490 (1953).
316. Kay, B. H., Pollitt, C. C., Fanning, I. D. & Hall, R. A. The experimental infection of horses with Murray Valley encephalitis and Ross River viruses. *Aust. Vet. J.* **64**, 52–55 (1987).

317. Miles, J. a. R. & Howes, D. W. Observations on virus encephalitis in South Australia. *Med. J. Aust.* **1**, 7–12 (1953).
318. Murray Valley encephalitis. *Victoria Department of Health Services* https://www2.health.vic.gov.au:443/public-health/infectious-diseases/disease-information-advice/murray-valley-encephalitis.
319. Murray Valley encephalitis - including symptoms, treatment and prevention. *Government of South Australia* https://www.sahealth.sa.gov.au/wps/wcm/connect/Public+Content/SA+Health+Internet/Conditions/Infectious+diseases/Murray+Valley+encephalitis/Murray+Valley+encephalitis+-+including+symptoms+treatment+and+prevention.
320. Russell, R. C. Population age composition and female longevity of the arbovirus vector Culex annulirostris skuse near Echuca, Victoria, in the Murray Valley of southeastern Australia 1979-1985. *Aust. J. Exp. Biol. Med. Sci.* **64 (Pt 6)**, 595–606 (1986).
321. Odend'hal, S. Murray Valley Encephalitis Virus. in *The Geographical Distribution of Animal Viral Diseases* (ed. Odend'hal, S.) 289–291 (Academic Press, 1983). doi:10.1016/B978-0-12-524180-9.50079-0.
322. Kunjin Virus Infection. *GIDEON - Global Infectious Diseases and Epidemiology Online Network* https://www.gideononline.com/2019/04/03/kunjin-virus-infection/ (2019).
323. Prow, N. A. The Changing Epidemiology of Kunjin Virus in Australia. *Int. J. Environ. Res. Public. Health* **10**, 6255–6272 (2013).
324. Ayoade, F. O. California Encephalitis: Background, Etiology, Epidemiology. *Medscape* https://emedicine.medscape.com/article/234159-overview (2016).
325. Eldridge, B. F., Glaser, C., Pedrin, R. E. & Chiles, R. E. The first reported case of California encephalitis in more than 50 years. *Emerg. Infect. Dis.* **7**, 451–452 (2001).
326. Young, P. R., Ng, L. F. P., Hall, R. A., Smith, D. W. & Johansen, C. A. 14 - Arbovirus Infections. in *Manson's Tropical Infectious Diseases (Twenty-third Edition)* (eds. Farrar, J. et al.) 129-161.e3 (W.B. Saunders, 2014). doi:10.1016/B978-0-7020-5101-2.00015-7.
327. Bewick, S., Agusto, F., Calabrese, J. M., Muturi, E. J. & Fagan, W. F. Epidemiology of La Crosse Virus Emergence, Appalachia Region, United States. *Emerg. Infect. Dis.* **22**, 1921–1929 (2016).
328. Szumlas, D. SEASONAL OCCURRENCE AND ABUNDANCE OF AEDES TRISERIATUS AND OTHER MOSQUITOES IN A LA CROSSE VIRUS-ENDEMIC AREA IN WESTERN NORTH CAROLINA. *Joumal Ameican Mosq. Control Assoc.* **12**, 184–193 (1996).
329. Foster, W. A. & Walker, E. D. 12 - MOSQUITOES (Culicidae). in *Medical and Veterinary Entomology* (eds. Mullen, G. & Durden, L.) 203–262 (Academic Press, 2002). doi:10.1016/B978-012510451-7/50014-1.
330. Amundson, T. E. & Yuill, T. M. Natural La Crosse virus infection in the red fox (Vulpes fulva), gray fox (urocyon cinereoargenteus), raccoon (Procyon lotor), and opossum (Didelphis virginiana). *Am. J. Trop. Med. Hyg.* **30**, 706–714 (1981).
331. Ellwanger, J., Kaminski, V. & Chies, J. Rocio virus: an overview. *Rev. Peru. Divulg. Científica En Genética Biol. Mol. RDGBM* **1**, 14–20 (2017).
332. Domingues, R. B. & Teixeira, A. L. Management of acute viral encephalitis in Brazil. *Braz. J. Infect. Dis. Off. Publ. Braz. Soc. Infect. Dis.* **13**, 433–439 (2009).
333. Stanford University. Stanford Humans and Viruses Database - Human Virology at Stanfrod - Virus Family List. https://web.stanford.edu/group/virus/viruslist.html (2015).
334. Mitchell, C. J., Monath, T. P. & Cropp, C. B. Experimental Transmission of Rocio Virus by Mosquitoes. *Am. J. Trop. Med. Hyg.* **30**, 465–472 (1981).

335. ECDC. Factsheet about tick-borne encephalitis (TBE). *European Centre for Disease Prevention and Control* https://www.ecdc.europa.eu/en/tick-borne-encephalitis/facts/factsheet (2019).
336. WHO & ECDC. *Tick-borne Encephalitis in Europe*. https://www.euro.who.int/__data/assets/pdf_file/0010/246169/Fact-sheet-Tick-borne-encephalitis-Eng.pdf (2014).
337. Foppa, I. M. The basic reproductive number of tick-borne encephalitis virus. An empirical approach. *J. Math. Biol.* **51**, 616–628 (2005).
338. Michelitsch, A., Wernike, K., Klaus, C., Dobler, G. & Beer, M. Exploring the Reservoir Hosts of Tick-Borne Encephalitis Virus. *Viruses* **11**, (2019).
339. Mansfield, K. L. *et al.* Tick-borne encephalitis virus - a review of an emerging zoonosis. *J. Gen. Virol.* **90**, 1781–1794 (2009).
340. WHO. *Global vector control response 2017–2030*. http://www.who.int/vector-control/publications/global-control-response/en/ (2017).
341. Bogovic, P. & Strle, F. Tick-borne encephalitis: A review of epidemiology, clinical characteristics, and management. *World J. Clin. Cases* **3**, 430–441 (2015).
342. Amicizia, D. *et al.* Epidemiology of tick-borne encephalitis (TBE) in Europe and its prevention by available vaccines. *Hum. Vaccines Immunother.* **9**, 1163–1171 (2013).
343. Grigoryeva, L. A. & Stanyukovich, M. K. Life cycle of the taiga tick Ixodes persulcatus (Acari: Ixodidae) in the North-West of Russia. *Exp. Appl. Acarol.* **69**, 347–357 (2016).
344. Rumyantsev, A. A. *et al.* Single-dose vaccine against tick-borne encephalitis. *Proc. Natl. Acad. Sci.* **110**, 13103–13108 (2013).
345. Norman, R., Bowers, R. G., Begon, M. & Hudson, P. J. Persistence of tick-borne virus in the presence of multiple host species: Tick reservoirs and parasite mediated competition. *J. Theor. Biol.* **200**, 111–118 (1999).
346. Jeffries, C. L. *et al.* Louping ill virus: an endemic tick-borne disease of Great Britain. *J. Gen. Virol.* **95**, 1005–1014 (2014).
347. Li, D., Ma, W. & Jiang, Z. An Epidemic Model for Tick-Borne Disease with Two Delays. *Journal of Applied Mathematics* vol. 2013 e427621 https://www.hindawi.com/journals/jam/2013/427621/ (2013).
348. Dagleish, M. P. Louping Ill in Animals - Nervous System. *Merck Veterinary Manual* https://www.merckvetmanual.com/nervous-system/louping-ill/louping-ill-in-animals (2020).
349. Spickler, A. *Louping Ill*. https://www.cfsph.iastate.edu/Factsheets/pdfs/louping_ill.pdf (2020).
350. Davidson, M., Williams, H. & Maclead, J. Louping ill in man: A forgotten disease. *J. Infect.* **23**, 241–249 (1991).
351. Kemenesi, G. & Bányai, K. Tick-Borne Flaviviruses, with a Focus on Powassan Virus. *Clin. Microbiol. Rev.* **32**, (2018).
352. Jacobsen, K. *The global prevalence of hepatitis A virus infection and susceptibility : a systematic review*. https://apps.who.int/iris/handle/10665/70180 (2009).
353. Fares, A. Seasonality of Hepatitis: A Review Update. *J. Fam. Med. Prim. Care* **4**, 96–100 (2015).
354. CDC. The ABCs of Hepatitis - for Health Professionals. *Center for Disease Control* https://www.cdc.gov/hepatitis/resources/professionals/pdfs/abctable.pdf (2020).
355. Pybus, O. G. *et al.* The epidemic behavior of the hepatitis C virus. *Science* **292**, 2323–2325 (2001).

356. Rein, D. B., Stevens, G. A., Theaker, J., Wittenborn, J. S. & Wiersma, S. T. The global burden of hepatitis E virus genotypes 1 and 2 in 2005. *Hepatol. Baltim. Md* **55**, 988–997 (2012).
357. Backer, J. Transmission dynamics of hepatitis E virus in pigs: Estimation from field data and effect of vaccination. *Epidemics* **4**, 86–92 (2012).
358. Pavio, N., Doceul, V., Bagdassarian, E. & Johne, R. Recent knowledge on hepatitis E virus in Suidae reservoirs and transmission routes to human. *Vet. Res.* **48**, 78 (2017).
359. Meng, X. J. Hepatitis E virus: Animal Reservoirs and Zoonotic Risk. *Vet. Microbiol.* **140**, 256 (2010).
360. Ashley, E. A. & White, N. J. The duration of Plasmodium falciparum infections. *Malar. J.* **13**, 500 (2014).
361. Abdel-Wahab, A. *et al.* Dynamics of gametocytes among Plasmodium falciparum clones in natural infections in an area of highly seasonal transmission. *J. Infect. Dis.* **185**, 1838–1842 (2002).
362. Smith, D. L., McKenzie, F. E., Snow, R. W. & Hay, S. I. Revisiting the Basic Reproductive Number for Malaria and Its Implications for Malaria Control. *PLOS Biol.* **5**, e42 (2007).
363. Gething, P. W. *et al.* A new world malaria map: Plasmodium falciparum endemicity in 2010. *Malar. J.* **10**, 378 (2011).
364. Snow, R. W., Guerra, C. A., Noor, A. M., Myint, H. Y. & Hay, S. I. The global distribution of clinical episodes of Plasmodium falciparum malaria. *Nature* **434**, 214–217 (2005).
365. Doolan, D. L., Dobaño, C. & Baird, J. K. Acquired immunity to malaria. *Clin. Microbiol. Rev.* **22**, 13–36, Table of Contents (2009).
366. Wongsrichanalai, C., Pickard, A. L., Wernsdorfer, W. H. & Meshnick, S. R. Epidemiology of drug-resistant malaria. *Lancet Infect. Dis.* **2**, 209–218 (2002).
367. WHO. *World malaria report 2017*. 196 http://www.who.int/malaria/publications/world-malaria-report-2017/report/en/ (2017).
368. Mendis, K., Sina, B. J., Marchesini, P. & Carter, R. The neglected burden of Plasmodium vivax malaria. *Am. J. Trop. Med. Hyg.* **64**, 97–106 (2001).
369. Howes, R. E. *et al.* Global Epidemiology of Plasmodium vivax. *Am. J. Trop. Med. Hyg.* **95**, 15–34 (2016).
370. Maguire, J. D. *et al.* Chloroquine-resistant Plasmodium malariae in south Sumatra, Indonesia. *Lancet Lond. Engl.* **360**, 58–60 (2002).
371. Mueller, I., Zimmerman, P. A. & Reeder, J. C. Plasmodium malariae and Plasmodium ovale--the ‘bashful’ malaria parasites. *Trends Parasitol.* **23**, 278–283 (2007).
372. Fuentes-Ramírez, A., Jiménez-Soto, M., Castro, R., Romero-Zuñiga, J. J. & Dolz, G. Molecular Detection of Plasmodium malariae/Plasmodium brasilianum in Non-Human Primates in Captivity in Costa Rica. *PLOS ONE* **12**, e0170704 (2017).
373. Collins, W. E. & Jeffery, G. M. Plasmodium malariae: Parasite and Disease. *Clin. Microbiol. Rev.* **20**, 579–592 (2007).
374. Collins, W. E. & Jeffery, G. M. Plasmodium ovale: parasite and disease. *Clin. Microbiol. Rev.* **18**, 570–581 (2005).
375. Sutherland, C. J. *et al.* Two nonrecombining sympatric forms of the human malaria parasite Plasmodium ovale occur globally. *J. Infect. Dis.* **201**, 1544–1550 (2010).
376. Brasil, P. *et al.* Outbreak of human malaria caused by Plasmodium simium in the Atlantic Forest in Rio de Janeiro: a molecular epidemiological investigation. *Lancet Glob. Health* **5**, e1038–e1046 (2017).

377. Goldman, I. F., Qari, S. H., Millet, P. G., Collins, W. E. & Lal, A. A. Circumsporozoite protein gene of Plasmodium simium, a Plasmodium vivax-like monkey malaria parasite. *Mol. Biochem. Parasitol.* **57**, 177–180 (1993).
378. Collins, W. E., Contacos, P. G., Guinn, E. G. & Skinner, J. C. Plasmodium simium in the Aotus trivirgatus monkey. *J. Parasitol.* **59**, 49–51 (1973).
379. Carlos, B. C., Rona, L. D. P., Christophides, G. K. & Souza-Neto, J. A. A comprehensive analysis of malaria transmission in Brazil. *Pathog. Glob. Health* **113**, 1–13 (2019).
380. Mumba, D. *et al.* Prevalence of Human African Trypanosomiasis in the Democratic Republic of the Congo. *PLoS Negl. Trop. Dis.* **5**, e1246 (2011).
381. Franco, J. R., Simarro, P. P., Diarra, A. & Jannin, J. G. Epidemiology of human African trypanosomiasis. *Clin. Epidemiol.* **6**, 257–275 (2014).
382. Fèvre, E. M., Wissmann, B. v, Welburn, S. C. & Lutumba, P. The Burden of Human African Trypanosomiasis. *PLoS Negl. Trop. Dis.* **2**, e333 (2008).
383. Funk, S., Nishiura, H., Heesterbeek, H., Edmunds, W. J. & Checchi, F. Identifying Transmission Cycles at the Human-Animal Interface: The Role of Animal Reservoirs in Maintaining Gambiense Human African Trypanosomiasis. *PLoS Comput. Biol.* **9**, e1002855 (2013).
384. Van den Bossche, P., de La Rocque, S., Hendrickx, G. & Bouyer, J. A changing environment and the epidemiology of tsetse-transmitted livestock trypanosomiasis. *Trends Parasitol.* **26**, 236–243 (2010).
385. Spickler, A. R. *African Animal Trypanosomiasis*. https://www.cfsph.iastate.edu/diseaseinfo/factsheets/ (2018).
386. Baker, N., de Koning, H. P., Mäser, P. & Horn, D. Drug resistance in African trypanosomiasis: the melarsoprol and pentamidine story. *Trends Parasitol.* **29**, (2013).
387. Njiokou, F. *et al.* Wild fauna as a probable animal reservoir for Trypanosoma brucei gambiense in Cameroon. *Infect. Genet. Evol.* **6**, 147–153 (2006).
388. Njiokou, F. *et al.* Domestic animals as potential reservoir hosts of Trypanosoma brucei gambiense in sleeping sickness foci in Cameroon. *Parasite* **17**, 61–66 (2010).
389. Campos, M. C. O., Leon, L. L., Taylor, M. C. & Kelly, J. M. Benznidazole-resistance in Trypanosoma cruzi: Evidence that distinct mechanisms can act in concert. *Mol. Biochem. Parasitol.* **193**, 17–19 (2014).
390. Campos, M. C. *et al.* Genome-wide mutagenesis and multi-drug resistance in American trypanosomes induced by the front-line drug benznidazole. *Sci. Rep.* **7**, 14407 (2017).
391. Cordovez, J. M., Rendon, L. M., Gonzalez, C. & Guhl, F. Using the basic reproduction number to assess the effects of climate change in the risk of Chagas disease transmission in Colombia. *Acta Trop.* **129**, 74–82 (2014).
392. Spickler, A. R. *American Trypanosomiasis*. https://www.cfsph.iastate.edu/diseaseinfo/factsheets/ (2017).
393. Jansen, A. M., Xavier, S. C. das C. & Roque, A. L. R. Trypanosoma cruzi transmission in the wild and its most important reservoir hosts in Brazil. *Parasit. Vectors* **11**, 502 (2018).
394. Fujita, O. *et al.* Animal reservoirs for Trypanosoma cruzi infection in an endemic area in Paraguay. *J. Vet. Med. Sci.* **56**, 305–308 (1994).
395. May, R. M. & Anderson, R. M. Population biology of infectious diseases: Part II. *Nature* **280**, 455–461 (1979).
396. Sokolow, S. H. *et al.* Reduced transmission of human schistosomiasis after restoration of a native river prawn that preys on the snail intermediate host. *Proc. Natl. Acad. Sci.* **112**, 9650–9655 (2015).

397. Sturrock, R. F. The Schistosomes and Their Intermediate Hosts. in *Schistosomiasis* vol. 3 7–83 (PUBLISHED BY IMPERIAL COLLEGE PRESS AND DISTRIBUTED BY WORLD SCIENTIFIC PUBLISHING CO., 2001).
398. Vale, N. *et al.* Praziquantel for Schistosomiasis: Single-Drug Metabolism Revisited, Mode of Action, and Resistance. *Antimicrob. Agents Chemother.* **61**, (2017).
399. Macpherson, C. N. L. & Craig, P. S. Animal reservoirs of schistosomiasis. in *Parasitic helminths and zoonoses in Africa* (eds. Macpherson, C. N. L. & Craig, P. S.) 224–236 (Springer Netherlands, 1991). doi:10.1007/978-94-011-3054-7_8.
400. Colley, D. G., Bustinduy, A. L., Secor, W. E. & King, C. H. Human schistosomiasis. *Lancet Lond. Engl.* **383**, 2253–2264 (2014).
401. Moen, L. & Tkacs, J. Schistosoma mansoni. *Animal Diversity Web* https://animaldiversity.org/accounts/Schistosoma_mansoni/ (2013).
402. Chernin, E. & Dunavan, C. A. The influence of host-parasite dispersion upon the capacity of Shistosoma mansoni miracidia to infect Australorbis glabratus. *Am. J. Trop. Med. Hyg.* **11**, 455–471 (1962).
403. Martins, A. V. Non-human vertebrate hosts of Schistosoma haematobium and Schistosoma mansoni. *Bull. World Health Organ.* **18**, 931–944 (1958).
404. Krishnamurthy, D., Katsikis, G., Bhargava, A. & Prakash, M. Schistosoma mansoni cercariae swim efficiently by exploiting an elastohydrodynamic coupling. *Nat. Phys.* **13**, 266–271 (2017).
405. Eveland, L. K. & Haseeb, M. A. Laboratory Rearing of Biomphalaria glabrata Snails and Maintenance of Larval Schistosomes In Vivo and In Vitro. in *Biomphalaria Snails and Larval Trematodes* (eds. Toledo, R. & Fried, B.) 33–55 (Springer, 2011). doi:10.1007/978-1-4419-7028-2_2.
406. Viana, M., Faust, C. L., Haydon, D. T., Webster, J. P. & Lamberton, P. H. L. The effects of subcurative praziquantel treatment on life-history traits and trade-offs in drug-resistant Schistosoma mansoni. *Evol. Appl.* **11**, 488–500 (2018).
407. Zhao, R. & Milner, F. A. A Mathematical model of Schistosoma mansoni in Biomphalaria glabrata with control strategies. *Bull. Math. Biol.* **70**, 1886–1905 (2008).
408. Coelho, P. M. Z. *et al.* Crab-eating Raccoon, Procyon cancrivorus nigripes (Mivart 1885) (Carnivora: Procyonidae) Naturally Infected with Schistosoma mansoni in Minas Gerais State, Brazil. *J. Parasitol.* **62**, 748–748 (1976).
409. Gao, S.-J. *et al.* The basic reproductive ratio of Barbour's two-host schistosomiasis model with seasonal fluctuations. *Parasit. Vectors* **10**, 42 (2017).
410. Coustau, C., Ataev, G., Jourdane, J. & Yoshino, T. P. Schistosoma japonicum: in vitro cultivation of miracidium to daughter sporocyst using a Biomphalaria glabrata embryonic cell line. *Exp. Parasitol.* **87**, 77–87 (1997).
411. He, Y.-X., Salafsky, B. & Ramaswamy, K. Host–parasite relationships of Schistosoma japonicum in mammalian hosts. *Trends Parasitol.* **17**, 320–324 (2001).
412. Li, Y. S. *et al.* Epidemiology of Schistosoma japonicum in China: morbidity and strategies for control in the Dongting Lake region. *Int. J. Parasitol.* **30**, 273–281 (2000).
413. Balen, J. *et al.* Prevalence, intensity and associated morbidity of Schistosoma japonicum infection in the Dongting Lake region, China. *Bull. World Health Organ.* **85**, 519–526 (2007).
414. Xie, F. *et al.* Life span and cercaria shedding of schistosome-infected snails in mountain region of Yunnan. *Zhongguo Ji Sheng Chong Xue Yu Ji Sheng Chong Bing Za Zhi* **8**, 4–7 (1990).
415. Crompton, D. W. T. How Much Human Helminthiasis Is There in the World? *J. Parasitol.* **85**, 397–403 (1999).

416. Larsson, B. *Three overviews on Environment and Aquaculture in the Tropics and Sub-tropics*. http://www.fao.org/3/AD002E/AD002E03.htm (1994).
417. Chitsulo, L., Engels, D., Montresor, A. & Savioli, L. The global status of schistosomiasis and its control. *Acta Trop.* **77**, 41–51 (2000).
418. Imbert-Establet, D., Moné, H., Tchuenté, L. A. T. & Jourdane, J. Permissiveness of two African wild rodents, Mastomys huberti and Arvicanthis niloticus, to Schistosoma intercalatum: epidemiological consequences. *Parasitol. Res.* **83**, 569–573 (1997).
419. Christensen, N. O., Mutani, A. & Frandsen, F. A review of the biology and transmission ecology of African bovine species of the genus Schistosoma. *Z. Parasitenkd. Berl. Ger.* **69**, 551–570 (1983).
420. Preston, J. M., Dargie, J. D. & MacLean, J. M. Patho-physiology of ovine schistosomiasis: I. A clinico-pathological study of experimental Schistosoma mattheei infections. *J. Comp. Pathol.* **83**, 401–415 (1973).
421. De Bont, J., Vercruysse, J., Sabbe, F., Southgate, V. R. & Rollinson, D. Schistosoma mattheei infections in cattle: changes associated with season and age. *Vet. Parasitol.* **57**, 299–307 (1995).
422. Davies, J. Schistosomiasis. *Medical Ecology* http://www.medicalecology.org/water/schistosomiasis/schistosomiasis.htm#sect3.5 (2014).
423. Bjørneboe, A. & Frandsen, F. A comparison of the characteristics of two strains of Schistosoma intercalatum Fisher, 1934 in mice. *J. Helminthol.* **53**, 195–203 (1979).
424. Hebner, R. ParaSite: Schistosoma Mekongi. *Stanford University* https://web.stanford.edu/group/parasites/ParaSites2009/RebeccaHebner_SchistoMekongi/RebeccaHebner_SchistoMekongi.htm (2009).
425. Jeyathilakan, N., Latha, B. R., Basith, S. A. & TANUVAS. Seasonal Prevalence of Schistosoma spindale in Ruminants at Chennai. *Tamil Nadu J. Vet. Anim. Sci.* **4**, 135–138 (2008).
426. Narain, K., Rajguru, S. K. & Mahanta, J. Incrimination of Schistosoma spindale as a causative agent of farmer's dermatitis in Assam with a note on liver pathology in mice. *J. Commun. Dis.* **30**, 1–6 (1998).
427. Kolárová, L., Skirnisson, K. & Horák, P. Schistosome cercariae as the causative agent of swimmer's itch in Iceland. *J. Helminthol.* **73**, 215–220 (1999).
428. U.S. Fish and Wildlife Service. *Rams Horn Snail (Indoplanorbis exustus)*. (2018).
429. Inder Singh, K., Krishnasamy, M., Ambu, S., Rasul, R. & Chong, N. L. Studies on animal schistosomes in Peninsular Malaysia: record of naturally infected animals and additional hosts of Schistosoma spindale. *Southeast Asian J. Trop. Med. Public Health* **28**, 303–307 (1997).
430. Bargues, M. D. *et al.* Lymnaea schirazensis, an Overlooked Snail Distorting Fascioliasis Data: Genotype, Phenotype, Ecology, Worldwide Spread, Susceptibility, Applicability. *PLOS ONE* **6**, e24567 (2011).
431. Blankespoor, H. & Reimink, R. The control of swimmer's itch in Michigan: past, present and future. *Mich. Acad.* **24**, 7–23 (1991).
432. Latif, B., Heo, C. C., Razuin, R., Shamalaa, D. V. & Tappe, D. Autochthonous Human Schistosomiasis, Malaysia. *Emerg. Infect. Dis.* **19**, 1340–1341 (2013).
433. Steinauer, M. L. *et al.* Interactions between natural populations of human and rodent schistosomes in the Lake Victoria region of Kenya: a molecular epidemiological approach. *PLoS Negl. Trop. Dis.* **2**, e222 (2008).
434. Morgan, J. A. T. *et al.* First Report of a Natural Hybrid between Schistosoma mansoni and S. rodhaini. *J. Parasitol.* **89**, 416–418 (2003).

435. Huyse, T. *et al.* Bidirectional Introgressive Hybridization between a Cattle and Human Schistosome Species. *PLOS Pathog.* **5**, e1000571 (2009).
436. Lengy, J. Studies on Schistosoma bovis (Sonsino, 1876) in Israel. I. Larval stages from egg to cercaria. *Bull. Res. Counc. Isr. Sect. E Exp. Med.* 1–36 (1962).
437. Raper, A. B. Schistosoma bovis infection in man. *East Afr. Med. J.* **28**, 50–54 (1951).
438. Oleaga, A. *et al.* Epidemiological surveillance of schistosomiasis outbreak in Corsica (France): Are animal reservoir hosts implicated in local transmission? *PLoS Negl. Trop. Dis.* **13**, e0007543 (2019).
439. Bates, P. A. Transmission of Leishmania metacyclic promastigotes by phlebotomine sand flies. *Int. J. Parasitol.* **37**, 1097–1106 (2007).
440. Lainson, R., Ryan, L. & Shaw, J. J. Infective stages of Leishmania in the sandfly vector and some observations on the mechanism of transmission. *Mem. Inst. Oswaldo Cruz* **82**, 421–424 (1987).
441. Pigott, D. M. *et al.* Global distribution maps of the leishmaniases. *eLife* **3**, e02851 (2014).
442. Rojas, R. *et al.* Resistance to Antimony and Treatment Failure in Human Leishmania (Viannia) Infection. *J. Infect. Dis.* **193**, 1375–1383 (2006).
443. Singh, S. P. *et al.* The epidemiology of Leishmania donovani infection in high transmission foci in India. *Trop. Med. Int. Health TM IH* **15 Suppl 2**, 12–20 (2010).
444. Tiwary, P. *et al.* Seasonal variation in the prevalence of sand flies infected with Leishmania donovani. *PloS One* **8**, e61370 (2013).
445. Torres-Guerrero, E., Quintanilla-Cedillo, M. R., Ruiz-Esmenjaud, J. & Arenas, R. Leishmaniasis: a review. *F1000Research* **6**, 750 (2017).
446. Piscopo, T. V. & Mallia Azzopardi, C. Leishmaniasis. *Postgrad. Med. J.* **83**, 649–657 (2007).
447. Ramalho-Ortigao, M., Saraiva, E. M. & Traub-Csekö, Y. M. Sand fly-Leishmania interactions: long relationships are not necessarily easy. *Open Parasitol. J.* **4**, 195–204 (2010).
448. Spickler, A. *Leishmaniasis (Cutaneous and Viscera)*. https://www.cfsph.iastate.edu/Factsheets/pdfs/leishmaniasis.pdf (2017).
449. Jambulingam, P., Pradeep Kumar, N., Nandakumar, S., Paily, K. P. & Srinivasan, R. Domestic dogs as reservoir hosts for Leishmania donovani in the southernmost Western Ghats in India. *Acta Trop.* **171**, 64–67 (2017).
450. Singh, N., Mishra, J., Singh, R. & Singh, S. Animal Reservoirs of Visceral Leishmaniasis in India. *J. Parasitol.* **99**, 64–67 (2013).
451. Lainson, R. & Rangel, E. F. Lutzomyia longipalpis and the eco-epidemiology of American visceral leishmaniasis, with particular reference to Brazil: a review. *Mem. Inst. Oswaldo Cruz* **100**, 811–827 (2005).
452. Shaw, J. J. Animal reservoirs of Leishmania in different ecological situations and their importance in the epidemiology of the disease. *Mem. Inst. Oswaldo Cruz* **83**, 486–490 (1988).
453. Rohousova, I. *et al.* Exposure to Leishmania spp. and sand flies in domestic animals in northwestern Ethiopia. *Parasit. Vectors* **8**, 360 (2015).
454. Amela, C. *et al.* Epidemiology of canine leishmaniasis in the Madrid region, Spain. *Eur. J. Epidemiol.* **11**, 157–161 (1995).
455. Quinnell, R. J., Courtenay, O., Garcez, L. & Dye, C. The epidemiology of canine leishmaniasis: transmission rates estimated from a cohort study in Amazonian Brazil. *Parasitology* **115 (Pt 2)**, 143–156 (1997).
456. Alemayehu, B. & Alemayehu, M. Leishmaniasis: A Review on Parasite, Vector and Reservoir Host. *Health Sci. J.* **11**, (2017).

457. Medkour, H. *et al.* Potential animal reservoirs (dogs and bats) of human visceral leishmaniasis due to Leishmania infantum in French Guiana. *PLoS Negl. Trop. Dis.* **13**, e0007456 (2019).
458. Echchakery, M. *et al.* Molecular detection of Leishmania infantum and Leishmania tropica in rodent species from endemic cutaneous leishmaniasis areas in Morocco. *Parasit. Vectors* **10**, (2017).
459. Roque, A. L. R. & Jansen, A. M. Wild and synanthropic reservoirs of Leishmania species in the Americas. *Int. J. Parasitol. Parasites Wildl.* **3**, 251–262 (2014).
460. Krayter, L., Schnur, L. F. & Schönian, G. The Genetic Relationship between Leishmania aethiopica and Leishmania tropica Revealed by Comparing Microsatellite Profiles. *PloS One* **10**, e0131227 (2015).
461. Baneth, G., Yasur-Landau, D., Gilad, M. & Nachum-Biala, Y. Canine leishmaniosis caused by Leishmania major and Leishmania tropica: comparative findings and serology. *Parasit. Vectors* **10**, 113 (2017).
462. Bogitsh, B. J., Carter, C. E. & Oeltmann, T. N. Chapter 6 - Blood and Tissue Protozoa I: Hemoflagellates. in *Human Parasitology (Fourth Edition)* (eds. Bogitsh, B. J., Carter, C. E. & Oeltmann, T. N.) 85–113 (Academic Press, 2013). doi:10.1016/B978-0-12-415915-0.00006-6.
463. Talmi-Frank, D. *et al.* Leishmania tropica in Rock Hyraxes (Procavia capensis) in a Focus of Human Cutaneous Leishmaniasis. *Am. J. Trop. Med. Hyg.* **82**, 814–818 (2010).
464. Pareyn, M. *et al.* Ecology and seasonality of sandflies and potential reservoirs of cutaneous leishmaniasis in Ochollo, a hotspot in southern Ethiopia. *PLoS Negl. Trop. Dis.* **13**, e0007667 (2019).
465. Anderson, J. M. *et al.* Seasonality and Prevalence of Leishmania major Infection in Phlebotomus duboscqi Neveu-Lemaire from Two Neighboring Villages in Central Mali. *PLoS Negl. Trop. Dis.* **5**, (2011).
466. Hamad, I. *et al.* Wild Gorillas as a Potential Reservoir of Leishmania major. *J. Infect. Dis.* **211**, 267–273 (2015).
467. Al-Tawfiq, J. A. & AbuKhamsin, A. Cutaneous leishmaniasis: a 46-year study of the epidemiology and clinical features in Saudi Arabia (1956-2002). *Int. J. Infect. Dis.* **8**, 244–250 (2004).
468. Dye, C., Guy, M. W., Elkins, D. B., Wilkes, T. J. & Killick-Kendrick, R. The life expectancy of phlebotomine sandflies: first field estimates from southern France. *Med. Vet. Entomol.* **1**, 417–425 (1987).
469. Corrêa, J. R., Brazil, R. P. & Soares, M. J. Leishmania (Viannia) lainsoni (Kinetoplastida: Trypanosomatidae), a divergent Leishmania of the Viannia subgenus: a mini review. *Mem. Inst. Oswaldo Cruz* **100**, 587–592 (2005).
470. Fagundes-Silva, G. A. *et al.* Leishmania (Viannia) naiffi: rare enough to be neglected? *Mem. Inst. Oswaldo Cruz* **110**, 797–800 (2015).
471. Coughlan, S. *et al.* Leishmania naiffi and Leishmania guyanensis reference genomes highlight genome structure and gene evolution in the Viannia subgenus. *R. Soc. Open Sci.* **5**, (2018).
472. Andrade-Narvaez, F. J. *et al.* Seasonal transmission of Leishmania (Leishmania) mexicana in the state of Campeche, Yucatan Peninsula, Mexico. *Mem. Inst. Oswaldo Cruz* **98**, 995–998 (2003).
473. Parnell, N. K., Guptill, L. & Solano-Gallego, L. CHAPTER 116 - Protozoal Infections. in *Handbook of Small Animal Practice (Fifth Edition)* (ed. Morgan, R. V.) 1132–1146 (W.B. Saunders, 2008). doi:10.1016/B978-1-4160-3949-5.50120-5.
474. Berzunza-Cruz, M. *et al.* Leishmania (L.) mexicana infected bats in Mexico: novel potential reservoirs. *PLoS Negl. Trop. Dis.* **9**, e0003438 (2015).

475. Muñoz-García, C. I. *et al.* The role of sloths and anteaters as Leishmania spp. reservoirs: a review and a newly described natural infection of Leishmania mexicana in the northern anteater. *Parasitol. Res.* **118**, 1095–1101 (2019).
476. Bonfante-Garrido, R. *et al.* Cutaneous leishmaniasis in cats (Felis domesticus) caused by Leishmania (Leishmania) venezuelensis. *Rev Cient* **6**, 187–190 (1996).
477. Walker, J. *et al.* Identification of developmentally-regulated proteins in Leishmania panamensis by proteome profiling of promastigotes and axenic amastigotes. *Mol. Biochem. Parasitol.* **147**, 64–73 (2006).
478. Ashford, R. W. Leishmaniasis reservoirs and their significance in control. *Clin. Dermatol.* **14**, 523–532 (1996).
479. Truppel, J. H. *et al.* Can Equids Be a Reservoir of Leishmania braziliensis in Endemic Areas? *PLOS ONE* **9**, e93731 (2014).
480. Dantas-Torres, F. Dogs as Reservoirs for Leishmania braziliensis. *Emerg. Infect. Dis.* **17**, 326–327 (2011).
481. Santos, F. J. A. *et al.* First Report of Canine Infection by Leishmania (Viannia) guyanensis in the Brazilian Amazon. *Int. J. Environ. Res. Public. Health* **17**, (2020).
482. Lainson, R., Shaw, J. J. & Póvoa, M. The importance of edentates (sloths and anteaters) as primary reservoirs of Leishmania braziliensis guyanensis, causative agent of “pianbois” in north Brazil. *Trans. R. Soc. Trop. Med. Hyg.* **75**, 611–612 (1981).
483. Llanos-Cuentas, E. A. *et al.* Natural infections of Leishmania peruviana in animals in the Peruvian Andes. *Trans. R. Soc. Trop. Med. Hyg.* **93**, 15–20 (1999).
484. Davies, C. R. *et al.* Cutaneous Leishmaniasis in the Peruvian Andes: Factors Associated with Variability in Clinical Symptoms, Response to Treatment, and Parasite Isolation Rate. *Clin. Infect. Dis.* **25**, 302–310 (1997).
485. Sacks, D. L. Leishmania-sand fly interactions controlling species-specific vector competence. Microreview. *Cell. Microbiol.* **3**, 189–196 (2001).
486. Sundar, S. & Chakravarty, J. An update on pharmacotherapy for leishmaniasis. *Expert Opin. Pharmacother.* **16**, 237–252 (2015).
487. McCarthy, J. Is anthelmintic resistance a threat to the program to eliminate lymphatic filariasis? *Am. J. Trop. Med. Hyg.* **73**, 232–233 (2005).
488. Fischer, P., Supali, T. & Maizels, R. M. Lymphatic filariasis and Brugia timori: prospects for elimination. *Trends Parasitol.* **20**, 351–355 (2004).
489. Manguin, S., Bangs, M. J., Pothikasikorn, J. & Chareonviriyaphap, T. Review on global co-transmission of human Plasmodium species and Wuchereria bancrofti by Anopheles mosquitoes. *Infect. Genet. Evol.* **10**, 159–177 (2010).
490. Dissanaike, A. S. Zoonotic aspects of filarial infections in man. *Bull. World Health Organ.* **57**, 349–357 (1979).
491. Anorital *et al.* Endemicity Brugia malayi Status Post Transmission Assessment Survey in Indonesia-2017. in *Advances in Health Sciences Research* vol. 22 (Atlantis Press, 2020).
492. WHO Expert Committee on Filariasis & World Health Organization. *Lymphatic filariasis : fourth report of the WHO Expert Committee on Filariasis [meeting held in Geneva from 31 October to 8 November 1983]*. https://apps.who.int/iris/handle/10665/39063 (1984).
493. Krishnamoorthy, K., Subramanian, S., Van Oortmarssen, G. J., Habbema, J. D. F. & Das, P. K. Vector survival and parasite infection: the effect of Wuchereria bancrofti on its vector Culex quinquefasciatus. *Parasitology* **129**, 43–50 (2004).

494. Renz, A. Studies on the dynamics of transmission of onchocerciasis in a Sudan-savanna area of North Cameroon III: Infection rates of the *Simulium* vectors and *Onchocerca volvulus* transmission potentials. *Ann. Trop. Med. Parasitol.* **81**, 239–252 (1987).
495. Osei-Atweneboana, M. Y. *et al.* Phenotypic Evidence of Emerging Ivermectin Resistance in Onchocerca volvulus. *PLoS Negl. Trop. Dis.* **5**, e998 (2011).
496. Brooker, S., Clements, A. C. A. & Bundy, D. A. P. Global Epidemiology, Ecology and Control of Soil-Transmitted Helminth Infections. in *Advances in Parasitology* vol. 62 221–261 (Elsevier, 2006).
497. McMillan, C. Simulium venustum. *Animal Diversity Web* https://animaldiversity.org/accounts/Simulium_venustum/ (2013).
498. Sciutto, E. *et al.* Development of the S3Pvac Vaccine Against Porcine *Taenia solium* Cysticercosis: A Historical Review. *J. Parasitol.* **99**, 686–692 (2013).
499. de Aluja, A. S. *et al.* Taenia solium cysticercosis: immunity in pigs induced by primary infection. *Vet. Parasitol.* **81**, 129–135 (1999).
500. DeGiorgio, C. M., Medina, M. T., Durón, R., Zee, C. & Escueta, S. P. Neurocysticercosis. *Epilepsy Curr.* **4**, 107–111 (2004).
501. Spickler, A. *Taeniasis, Cysticercosis and Coenurosis*. https://www.cfsph.iastate.edu/Factsheets/pdfs/taenia.pdf (2020).
502. McManus, D. P., Zhang, W., Li, J. & Bartley, P. B. Echinococcosis. *The Lancet* **362**, 1295–1304 (2003).
503. Liu, J., Liu, L., Feng, X. & Feng, J. Global dynamics of a time-delayed echinococcosis transmission model. *Adv. Differ. Equ.* **2015**, 99 (2015).
504. Moro, P. & Schantz, P. M. Echinococcosis: a review. *Int. J. Infect. Dis.* **13**, 125–133 (2009).
505. Spickler, A. *Echinococcosis*. https://www.cfsph.iastate.edu/Factsheets/pdfs/echinococcosis.pdf (2020).
506. Chaligiannis, I. *et al.* Echinococcus granulosus infection dynamics in livestock of Greece. *Acta Trop.* **150**, 64–70 (2015).
507. Robertson, L. J. Parasites in Food: Occurrence and Detection. in *Encyclopedia of Food and Health* (eds. Caballero, B., Finglas, P. M. & Toldrá, F.) 219–224 (Academic Press, 2016). doi:10.1016/B978-0-12-384947-2.00518-3.
508. Torgerson, P. R., Keller, K., Magnotta, M. & Ragland, N. The Global Burden of Alveolar Echinococcosis. *PLoS Negl. Trop. Dis.* **4**, e722 (2010).
509. Veit, P. *et al.* Influence of environmental factors on the infectivity of *Echinococcus multilocularis* eggs. *Parasitology* **110**, 79–86 (1995).
510. Webster, G. A. & Cameron, T. W. M. OBSERVATIONS ON EXPERIMENTAL INFECTIONS WITH ECHINOCOCCUS IN RODENTS. *Can. J. Zool.* **39**, 877–891 (1961).
511. Chan, M. & Johansson, M. A. The Incubation Periods of Dengue Viruses. *PLoS ONE* **7**, e50972 (2012).
512. Grassly, N. C., Ward, M. E., Ferris, S., Mabey, D. C. & Bailey, R. L. The Natural History of Trachoma Infection and Disease in a Gambian Cohort with Frequent Follow-Up. *PLoS Negl. Trop. Dis.* **2**, e341 (2008).
513. Gershman, M. D. & Staples, J. E. Chapter 4: Travel-Related Infectious Diseases | Yellow Fever. in *CDC Health Information for International Travel 2020 - The Yellow Book* vol. 23 (CDC, 2019).
514. Hanley, K. A. *et al.* Fever versus fever: The role of host and vector susceptibility and interspecific competition in shaping the current and future distributions of the sylvatic cycles of dengue virus and yellow fever virus. *Infect. Genet. Evol.* **19**, 292–311 (2013).

515. Vasconcelos, P. F. C. & Monath, T. P. Yellow Fever Remains a Potential Threat to Public Health. *Vector-Borne Zoonotic Dis.* **16**, 566–567 (2016).
516. Massad, E., Coutinho, F. A., Burattini, M. N. & Lopez, L. F. The risk of yellow fever in a dengue-infested area. *Trans. R. Soc. Trop. Med. Hyg.* **95**, 370–374 (2001).
517. Monath, T. P. Yellow fever: an update. *Lancet Infect. Dis.* **1**, 11–20 (2001).
518. Johansson, M. A., Arana-Vizcarrondo, N., Biggerstaff, B. J. & Staples, J. E. Incubation periods of Yellow fever virus. *Am. J. Trop. Med. Hyg.* **83**, 183–188 (2010).
519. George, D. B. *et al.* Host and viral ecology determine bat rabies seasonality and maintenance. *Proc. Natl. Acad. Sci.* **108**, 10208–10213 (2011).
520. Turmelle, A. S., Jackson, F. R., Green, D., McCracken, G. F. & Rupprecht, C. E. Host immunity to repeated rabies virus infection in big brown bats. *J. Gen. Virol.* **91**, 2360–2366 (2010).
521. Hampson, K. *et al.* Estimating the Global Burden of Endemic Canine Rabies. *PLoS Negl. Trop. Dis.* **9**, e0003709 (2015).
522. Spickler, A. *Rabies and Rabies-Related Lyssaviruses*. https://www.cfsph.iastate.edu/Factsheets/pdfs/rabies.pdf (2012).
523. Shalaby, H. A., Abdel-Shafy, S. & Derbala, A. A. The role of dogs in transmission of Ascaris lumbricoides for humans. *Parasitol. Res.* **106**, 1021–1026 (2010).
524. Obafemi Awolowo University, Ife-Ife, Asaolu, S. O., Ofoezie, I. E., & Obafemi Awolowo University, Ife-Ife. Ascaris spp. in *Global Water Pathogen Project* (Michigan State University, 2019). doi:10.14321/waterpathogens.41.
525. Stuart, M. D., Greenspan, L. L., Glander, K. E. & Clarke, M. R. A Coprological Survey of Parasites of Wild Mantled Howling Monkeys, Alouatta palliata palliata. *J. Wildl. Dis.* **26**, 547–549 (1990).
526. Bradley, J. E. & Jackson, J. A. Immunity, immunoregulation and the ecology of trichuriasis and ascariasis. *Parasite Immunol.* **26**, 429–441 (2004).
527. Spickler, A. *Trichuriasis*. https://www.cfsph.iastate.edu/Factsheets/pdfs/trichuriasis.pdf (2019).
528. Pullan, R. L., Smith, J. L., Jasrasaria, R. & Brooker, S. J. Global numbers of infection and disease burden of soil transmitted helminth infections in 2010. *Parasit. Vectors* **7**, 1–19 (2014).
529. Izurieta, R., Reina-Ortiz, M. & Ochoa-Capello, T. Trichuris trichiura. in *Global Water Pathogen Project* (UNESCO, 2018).
530. Hotez, P. J., Bethony, J., Bottazzi, M. E., Brooker, S. & Buss, P. Hookworm: "The Great Infection of Mankind". *PLoS Med.* **2**, e67 (2005).
531. Pritchard, D. I., Quinnell, R. J. & Walsh, E. A. Immunity in humans to Necator americanus-. IgE, parasite weight and fecundity. *Parasite Immunol.* **17**, 71–75 (1995).
532. Bradley, M., Chandiwana, S. K., Bundy, D. A. & Medley, G. F. The epidemiology and population biology of Necator americanus infection in a rural community in Zimbabwe. *Trans. R. Soc. Trop. Med. Hyg.* **86**, 73–76 (1992).
533. Anderson, R. M. & May, R. M. Population dynamics of human helminth infections: control by chemotherapy. *Nature* **297**, 557–563 (1982).
534. Kimberlin, D. W., Brady, M. T., Jackson, M. A. & Long, S. S. Section 3: Summaries of Infectious Diseases | Hookworm Infections. in *Red Book 2018* 453–454 (American Academy of Pediatrics, 2018).
535. Boyko, R. H. *et al.* Dogs and pigs are transport hosts of Necator americanus: Molecular evidence for a zoonotic mechanism of human hookworm transmission in Ghana. *Zoonoses Public Health* **67**, 474–483 (2020).

536. Changhua, L. *et al.* Epidemiology of human hookworm infections among adult villagers in Hejiang and Santai Counties, Sichuan Province, China. *Acta Trop.* **73**, 243–249 (1999).
537. Spickler, A. *Zoonotic Hookworms*. https://www.cfsph.iastate.edu/Factsheets/pdfs/hookworms.pdf (2013).
538. Rogers, L. L. & Rogers, S. M. Parasites of Bears: A Review. *Bears Their Biol. Manag.* **3**, 411 (1976).
539. Asakawa, M., Gardner, S. & Mano, T. Research Note: First Record of Ancylostoma malayanum (Alessandrini, 1905) from Brown Bears (Ursus arctos L.). *Comp. Parasitoloty* **73**, 282–284 (2006).
540. Setasuban, P. & Vajrasthira, S. Ancylostoma malayanum, Alessandrini, 1905 in Thailand. *Southeast Asian J. Trop. Med. Public Health* **6**, 505–509 (1975).
541. Traub, R. J. Ancylostoma ceylanicum, a re-emerging but neglected parasitic zoonosis. *Int. J. Parasitol.* **43**, 1009–1015 (2013).
542. Speare, R., Bradbury, R. S. & Croese, J. A Case of Ancylostoma ceylanicum Infection Occurring in an Australian Soldier Returned from Solomon Islands. *Korean J. Parasitol.* **54**, 533–536 (2016).
543. Carroll, S. M. & Grove, D. I. Parasitological, hematologic, and immunologic responses in acute and chronic infections of dogs with Ancylostoma ceylanicum: a model of human hookworm infection. *J. Infect. Dis.* **150**, 284–294 (1984).
544. Inpankaew, T. *et al.* High prevalence of Ancylostoma ceylanicum hookworm infections in humans, Cambodia, 2012. *Emerg. Infect. Dis.* **20**, 976–982 (2014).
545. Bethony, J. *et al.* Soil-transmitted helminth infections: ascariasis, trichuriasis, and hookworm. *Lancet Lond. Engl.* **367**, 1521–1532 (2006).
546. Hoagland, K. E. & Schad, G. A. Necator americanus and Ancylostoma duodenale: Life history parameters and epidemiological implications of two sympatric hookworms of humans. *Exp. Parasitol.* **44**, 36–49 (1978).
547. Anderson, R., Truscott, J. & Hollingsworth, T. D. The coverage and frequency of mass drug administration required to eliminate persistent transmission of soil-transmitted helminths. *Philos. Trans. R. Soc. B Biol. Sci.* **369**, 20130435 (2014).
548. Strait, K., Else, J. G. & Eberhard, M. L. Chapter 4 - Parasitic Diseases of Nonhuman Primates. in *Nonhuman Primates in Biomedical Research (Second Edition)* (eds. Abee, C. R., Mansfield, K., Tardif, S. & Morris, T.) 197–297 (Academic Press, 2012). doi:10.1016/B978-0-12-381366-4.00004-3.
549. Patel, S., Aboutalebi, S., Vindhya, P. L. & Smith, J. What's eating you? Extensive cutaneous larva migrans (Ancylostoma braziliense). *Cutis* **82**, 239–240 (2008).
550. AAVP. Ancylostoma braziliense. *American Association of Veterinary Parasitologists* https://www.aavp.org/wiki/nematodes/strongylida/ancylostomatoidea/ancylostoma-braziliense/.
551. Norris, D. E. The Migratory Behavior of the Infective-Stage Larvae of Ancylostoma braziliense and Ancylostoma tubaeforme in Rodent Paratenic Hosts. *J. Parasitol.* **57**, 998–1009 (1971).
552. Miller, T. A. Transfer of immunity to Ancylostoma caninum infection in pups by serum and lymphoid cells. *Immunology* **12**, 231–241 (1967).
553. Little, M. D. Observations on the Possible Role of Insects as Paratenic Hosts for Ancylostoma caninum. *J. Parasitol.* **47**, 263–267 (1961).
554. Barr, S. & Irwin, P. Vetlexicon Canis | Ancylostoma caninum in dogs. *Vetstream* https://www.vetstream.com/treat/canis/bug/ancylostoma-caninum (2020).
555. Saeed, S. Ancylostoma caninum. *Animal Diversity Web* https://animaldiversity.org/accounts/Ancylostoma_caninum/ (2003).

556. Ngui, R., Lim, Y. A. L., Traub, R., Mahmud, R. & Mistam, M. S. Epidemiological and Genetic Data Supporting the Transmission of Ancylostoma ceylanicum among Human and Domestic Animals. *PLoS Negl. Trop. Dis.* **6**, (2012).
557. Beknazarova, M., Whiley, H. & Ross, K. Advocating for both Environmental and Clinical Approaches to Control Human Strongyloidiasis. *Pathogens* **5**, 59 (2016).
558. Page, W., Judd, J. A. & Bradbury, R. S. The Unique Life Cycle of Strongyloides stercoralis and Implications for Public Health Action. *Trop. Med. Infect. Dis.* **3**, (2018).
559. White, M. A. F., Whiley, H. & Ross, K. E. A Review of Strongyloides spp. Environmental Sources Worldwide. *Pathog. Basel Switz.* **8**, (2019).
560. Burkhart, C. N. & Burkhart, C. G. Assessment of frequency, transmission, and genitourinary complications of enterobiasis (pinworms): Pinworms. *Int. J. Dermatol.* **44**, 837–840 (2005).
561. Kubiak, K., Dzika, E. & Paukszto, Ł. Enterobiasis epidemiology and molecular characterization of Enterobius vermicularis in healthy children in north-eastern Poland. *Helminthologia* **54**, 284–291 (2017).
562. Wendt, S. *et al.* The Diagnosis and Treatment of Pinworm Infection. *Dtsch. Ärztebl. Int.* **116**, 213–219 (2019).
563. Des Clers, S. A. & Wootten, R. Modelling the population dynamics of the sealworm Pseudoterranova decipiens. *Neth. J. Sea Res.* **25**, 291–299 (1990).
564. Hochberg, N. S. & Hamer, D. H. Anisakidosis: Perils of the Deep. *Clin. Infect. Dis.* **51**, 806–812 (2010).
565. Audicana, M., Pozo, M. D., Iglesias, R. & Ubeira, F. Anisakis simplex and Pseudoterranova decipiens. *Int. Handb. Foodborne Pathog.* (2003).
566. Ramanan, P., Blumberg, A. K., Mathison, B. & Pritt, B. S. Parametrial Anisakidosis. *J. Clin. Microbiol.* **51**, 3430–3434 (2013).
567. Anderson, R. C. *Nematode parasites of vertebrates: their development and transmission*. (CABI Pub., 2000).
568. Aibinu, I. E., Smooker, P. M. & Lopata, A. L. Anisakis Nematodes in Fish and Shellfish- from infection to allergies. *Int. J. Parasitol. Parasites Wildl.* **9**, 384–393 (2019).
569. Pearson, R. D. Anisakiasis - Infectious Diseases. *Merck Manuals Professional Edition* https://www.merckmanuals.com/professional/infectious-diseases/nematodes-roundworms/anisakiasis (2020).
570. *Food-borne parasitic zoonoses: fish and plant-borne parasites*. (Springer, 2007).
571. Saichua, P., Nithikathkul, C. & Kaewpitoon, N. Human intestinal capillariasis in Thailand. *World J. Gastroenterol.* **14**, 506 (2008).
572. Cross, J. H. & Basaca-Sevilla, V. Capillariasis philippinensis: a fish-borne parasitic zoonosis. *Southeast Asian J. Trop. Med. Public Health* **22 Suppl**, 153–157 (1991).
573. Cross, J. H. *et al.* Studies on the experimental transmission of Capillaria philippinensis in monkeys*. *Trans. R. Soc. Trop. Med. Hyg.* **66**, 819–827 (1972).
574. Bhaibulaya, M. & Indra-Ngarm, S. Amaurornis phoenicurus and Ardeola bacchus as experimental definitive hosts for Capillaria philippinensis in Thailand. *Int. J. Parasitol.* **9**, 321–322 (1979).
575. El-Dib, N. A., El-Badry, A. A., Ta-Tang, T.-H. & Rubio, J. M. Molecular detection of Capillaria philippinensis: An emerging zoonosis in Egypt. *Exp. Parasitol.* **154**, 127–133 (2015).
576. Cross, J. H. Intestinal capillariasis. *Parasitol. Today* **6**, 26–28 (1990).
577. Gill, J. H. & Lacey, E. Avermectin\milbemycin resistance in trichostrongyloid nematodes. *Int. J. Parasitol.* **28**, 863–877 (1998).

578. Altizer, S. *et al.* Seasonality and the dynamics of infectious diseases. *Ecol. Lett.* **9**, 467–484 (2006).
579. Buonfrate, D. *et al.* Four clusters of Trichostrongylus infection diagnosed in a single center, in Italy. *Infection* **45**, 233–236 (2017).
580. Audebert, F., Hoste, H. & Durette-Desset, M. C. Life cycle of Trichostrongylus retortaeformis in its natural host, the rabbit (Oryctolagus cuniculus). *J. Helminthol.* **76**, 189–192 (2002).
581. Bundy, D. A., Cooper, E. S. & Brooker, S. Nematodes Limited to the Intestinal Tract (Enterobius vermicularis, Trichuris trichiura, Capillaria philippinensis and Trichostrongylus spp.). in *Hunter's Tropical Medicine and Emerging Infectious Disease* 797–803 (Elsevier, 2013). doi:10.1016/B978-1-4160-4390-4.00107-7.
582. Stanford University. Stanford ParaSites Database - Parasites and Pestilence. https://web.stanford.edu/group/parasites/ (2010).
583. Shaw, J. L. & Moss, R. The role of parasite fecundity and longevity in the success of *Trichostrongylus tenuis* in low density red grouse populations. *Parasitology* **99**, 253–258 (1989).
584. Ranjbar, M. J. *et al.* Helminth Infections of Rodents and Their Zoonotic Importance in Boyer-Ahmad District, Southwestern Iran. *Iran. J. Parasitol.* **12**, 572–579 (2017).
585. Chapter 2 - Nematoda. in *Parasiticide Screening, Volume 2* (eds. Marchiondo, A. A., Cruthers, L. R. & Fourie, J. J.) vol. 2 135–335 (Academic Press, 2019).
586. Hoste, H., Mallet, S. & Koch, C. Trichostrongylus colubriformis infection in rabbits: persistence of the distal adaptive response to parasitism after anthelmintic treatment. *J. Comp. Pathol.* **113**, 145–153 (1995).
587. Diemert, D. J. 365 - Intestinal Nematode Infections. in *Goldman's Cecil Medicine (Twenty Fourth Edition)* (eds. Goldman, L. & Schafer, A. I.) vol. 2 2064–2068 (W.B. Saunders, 2012).
588. Wang, Q.-P., Wu, Z.-D., Wei, J., Owen, R. L. & Lun, Z.-R. Human Angiostrongylus cantonensis: an update. *Eur. J. Clin. Microbiol. Infect. Dis.* **31**, 389–395 (2012).
589. Mackerras, M. & Sandars, D. The life history of the rat lung-worm, Angiostrongylus cantonensis (Chen) (Nematoda: Metastrongylidae). *Aust. J. Zool.* **3**, 1 (1955).
590. Lv, S. *et al.* The emergence of angiostrongyliasis in the People's Republic of China: the interplay between invasive snails, climate change and transmission dynamics. *Freshw. Biol.* **56**, 717–734 (2011).
591. Rodriguez, R. *et al.* Dogs may be a reservoir host for Angiostrongylus costaricensis. *Rev. Inst. Med. Trop. São Paulo* **44**, 55–56 (2002).
592. Pozio, E. 8 - Foodborne nematodes. in *Foodborne Parasites in the Food Supply Web* (ed. Gajadhar, A. A.) 165–199 (Woodhead Publishing, 2015). doi:10.1016/B978-1-78242-332-4.00008-4.
593. Romero-Alegría, A. *et al.* Angiostrongylus costaricensis: Systematic Review of Case Reports. *Adv. Infect. Dis.* **4**, (2014).
594. Kai, M. *et al.* Analysis of Drug-Resistant Strains of Mycobacterium leprae in an Endemic Area of Vietnam. *Clin. Infect. Dis.* **52**, e127–e132 (2011).
595. Mohanty, P. *et al.* Viability of Mycobacterium leprae in the environment and its role in leprosy dissemination. *Indian J. Dermatol. Venereol. Leprol.* **82**, 23 (2016).
596. Truman, R. W., Kumaresan, J. A., McDonough, C. M., Job, C. K. & Hastings, R. C. Seasonal and spatial trends in the detectability of leprosy in wild armadillos. *Epidemiol. Infect.* **106**, 549–560 (1991).
597. Wheat, W. H. *et al.* Long-term Survival and Virulence of Mycobacterium leprae in Amoebal Cysts. *PLoS Negl. Trop. Dis.* **8**, e3405 (2014).

598. Truman, R. W. *et al.* The Armadillo as a Model for Peripheral Neuropathy in Leprosy. *ILAR J.* **54**, 304–314 (2014).
599. Ploemacher, T., Faber, W. R., Menke, H., Rutten, V. & Pieters, T. Reservoirs and transmission routes of leprosy; A systematic review. *PLoS Negl. Trop. Dis.* **14**, e0008276 (2020).
